# Complete genome sequence, lifestyle, and multi-drug resistance of the human pathogen *Corynebacterium resistens *DSM 45100 isolated from blood samples of a leukemia patient

**DOI:** 10.1186/1471-2164-13-141

**Published:** 2012-04-23

**Authors:** Jasmin Schröder, Irena Maus, Katja Meyer, Stephanie Wördemann, Jochen Blom, Sebastian Jaenicke, Jessica Schneider, Eva Trost, Andreas Tauch

**Affiliations:** 1 Institut für Genomforschung und Systembiologie, Centrum für Biotechnologie, Universität Bielefeld, Universitätsstraße 27, D-33615 Bielefeld, Germany; 2 Bioinformatics Resource Facility, Centrum für Biotechnologie, Universität Bielefeld, Universitätsstraße 27, D-33615 Bielefeld, Germany; 3 CLIB Graduate Cluster Industrial Biotechnology, Centrum für Biotechnologie, Universität Bielefeld, Universitätsstraße 27, D-33615 Bielefeld, Germany

## Abstract

**Background:**

*Corynebacterium resistens *was initially recovered from human infections and recognized as a new coryneform species that is highly resistant to antimicrobial agents. Bacteremia associated with this organism in immunocompromised patients was rapidly fatal as standard minocycline therapies failed. *C. resistens *DSM 45100 was isolated from a blood culture of samples taken from a patient with acute myelocytic leukemia. The complete genome sequence of *C. resistens *DSM 45100 was determined by pyrosequencing to identify genes contributing to multi-drug resistance, virulence, and the lipophilic lifestyle of this newly described human pathogen.

**Results:**

The genome of *C. resistens *DSM 45100 consists of a circular chromosome of 2,601,311 bp in size and the 28,312-bp plasmid pJA144188. Metabolic analysis showed that the genome of *C. resistens *DSM 45100 lacks genes for typical sugar uptake systems, anaplerotic functions, and a fatty acid synthase, explaining the strict lipophilic lifestyle of this species. The genome encodes a broad spectrum of enzymes ensuring the availability of exogenous fatty acids for growth, including predicted virulence factors that probably contribute to fatty acid metabolism by damaging host tissue. *C. resistens *DSM 45100 is able to use external L-histidine as a combined carbon and nitrogen source, presumably as a result of adaptation to the hitherto unknown habitat on the human skin. Plasmid pJA144188 harbors several genes contributing to antibiotic resistance of *C. resistens *DSM 45100, including a tetracycline resistance region of the Tet W type known from *Lactobacillus reuteri *and *Streptococcus suis*. The *tet*(W) gene of pJA144188 was cloned in *Corynebacterium glutamicum *and was shown to confer high levels of resistance to tetracycline, doxycycline, and minocycline *in vitro*.

**Conclusions:**

The detected gene repertoire of *C. resistens *DSM 45100 provides insights into the lipophilic lifestyle and virulence functions of this newly recognized pathogen. Plasmid pJA144188 revealed a modular architecture of gene regions that contribute to the multi-drug resistance of *C. resistens *DSM 45100. The *tet*(W) gene encoding a ribosomal protection protein is reported here for the first time in corynebacteria. Cloning of the *tet*(W) gene mediated resistance to second generation tetracyclines in *C. glutamicum*, indicating that it might be responsible for the failure of minocycline therapies in patients with *C. resistens *bacteremia.

## Background

The genus *Corynebacterium *belongs to the taxonomic class *Actinobacteria *and represents a diverse group of Gram-positive bacteria with a DNA of high G + C content, whose members were recognized in a large variety of habitats [1]. The most prominent species of the genus *Corynebacterium *is the human pathogen *Corynebacterium diphtheriae*, which is the etiological agent of the acute, communicable disease diphtheria [2]. With the exception of *C. diphtheriae*, the pathogenicity of other corynebacterial species from clinical sources has been underestimated for a long time, as they were often regarded as skin contaminants in human infections [3]. The improved taxonomic recognition of corynebacteria in clinical specimens and the increasing number of case reports associating non-diphtherial species with infections in humans and also in animals has changed this view during the last decade [4,5]. In particular, the common skin colonizers *Corynebacterium urealyticum *and *Corynebacterium jeikeium*, which both belong to a separate branch in the phylogenetic tree of the genus *Corynebacterium *[6], were frequently associated with infections in immunocompromised patients. *C. urealyticum *is primarily recovered from hospitalized elderly individuals and can cause urinary tract infections [7], whereas *C. jeikeium *is associated with a variety of nosocomial infections, for instance with endocarditis after cardiac surgery and with bacteremia in hematological patients [8,9]. The majority of clinical isolates assigned to these species displayed a remarkable multi-drug resistance in such a way that only glycopeptide antibiotics remain universally active against these pathogens [10,11]. The development of multi-drug resistance in corynebacteria is probably enhanced by the selective pressure occurring in the hospital setting and has tremendous consequences for the successful treatment of human infections, especially in elderly individuals and in immunocompromised patients [12,13].

In 2005, a new multi-drug resistant corynebacterium was isolated from human infections in Japan and named *Corynebacterium resistens *[14]. Five strains of this bacterium were recovered from blood samples, bronchial aspirates, and abscess specimens and characterized by measuring their susceptibilities to antimicrobial agents. Four strains were obtained from inpatients and revealed high levels of resistance to macrolides, aminoglycosides, tetracyclines, quinolones, and β-lactams, whereas the fifth isolate was recovered from an outpatient and shown to be susceptible to imipenem and minocycline. The glycopeptides vancomycin and teicoplanin remained universally active against the five isolates. Although the administration of vancomycin is generally regarded as the first choice to eradicate multi-drug resistant corynebacteria, the use of this glycopeptide antibiotic is restricted to methicillin-resistant *Staphylococcus aureus *(MRSA) in Japan. Minocycline, a second generation tetracycline [15], was administered instead, but this antimicrobial therapy failed and probably contributed to the subsequent death of a patient from sepsis [14].

Experimental data from a polyphasic taxonomic approach revealed that the five clinical isolates were genetically identical and repesent a new subline within the genus *Corynebacterium*, with the multi-drug resistant species *C. urealyticum *and *C. jeikeium *as phylogenetic neighbors [14]. The type strain of this new corynebacterial species is *C. resistens *DSM 45100 (originally referred to as SICGH 158) that was isolated from a positive blood culture of samples taken from a patient with acute myelocytic leukemia [14]. In this study, we present the complete genome sequence and bioinformatic analysis of *C. resistens *DSM 45100 providing detailed insights into the lipophilic lifestyle and the virulence factors of this strain. During the sequencing project we recognized that *C. resistens *DSM 45100 harbors a plasmid that we named pJA144188. The DNA sequences of the chromosome and pJA144188 revealed the molecular mechanisms leading to the extensive antibiotic resistance of *C. resistens *DSM 45100. We detected the *tet*(W) gene to cause resistance to minocycline and verified its functioning in corynebacteria by expressing the resistance determinant in the susceptible host strain *Corynebacterium glutamicum *ATCC 13032.

## Results and discussion

### Pyrosequencing and annotation of the *C. resistens *DSM 45100 genome

**Figure 1:**
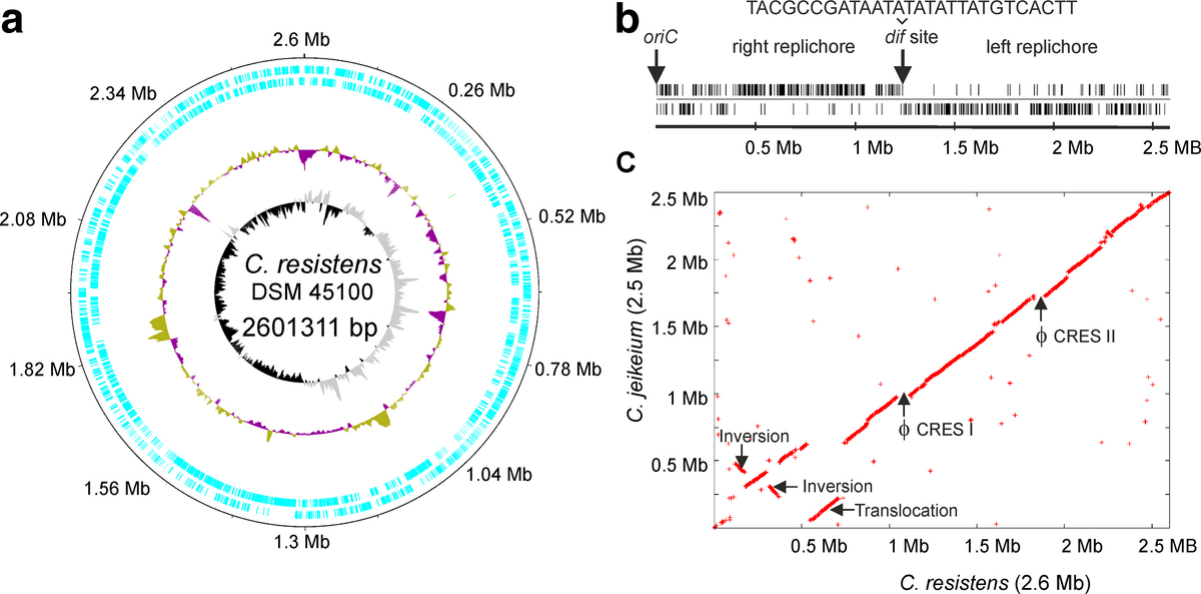
**Features of the *C. resistens *DSM 45100 chromosome**. **(A)**, Circular representation of the annotated chromosome of *C. resistens *DSM 45100. The circles represent from the outside: circle 1, DNA base position [Mb]; circle 2, protein-coding regions transcribed clockwise; circle 3, protein-coding regions transcribed anticlockwise; circle 4, G + C content plotted using a 10-kb window; circle 5, G/C skew plotted using a 10-kb window. The plot was generated with the web version of the DNAPlotter tool. **(B)**, Distribution of architecture imparting sequences in the *C. resistens *DSM 45100 chromosome. The distribution of the octamers G(A/T/C)GGGGGA and (T/C)GGGGGAG on the leading and lagging strands of the chromosome is shown. The origin of chromosomal replication (*oriC*) is marked. The deduced *dif *locus is located at around 1.23 Mbp of the chromosomal map. The sequence of the 28-bp *dif *site is shown. **(C)**, Synteny plot between the chromosomes of *C. resistens *DSM 45100 and *C. jeikeium *K411. The X-Y plot shows dots forming syntenic regions between the two chromosomes. Each dot represents a *C. resistens *protein having an ortholog in the *C. jeikeium *genome, with co-ordinates corresponding to the position of the respective coding region in each genome. The orthologs were identified by reciprocal best BLASTP matches using the predicted amino acid sequences of *C. resistens *proteins. The detected genomic rearrangements are labeled; the positions of the prophages ΦCRES I and ΦCRES II in the chromosome of *C. resistens *DSM 45100 are marked.

**Figure 2:**
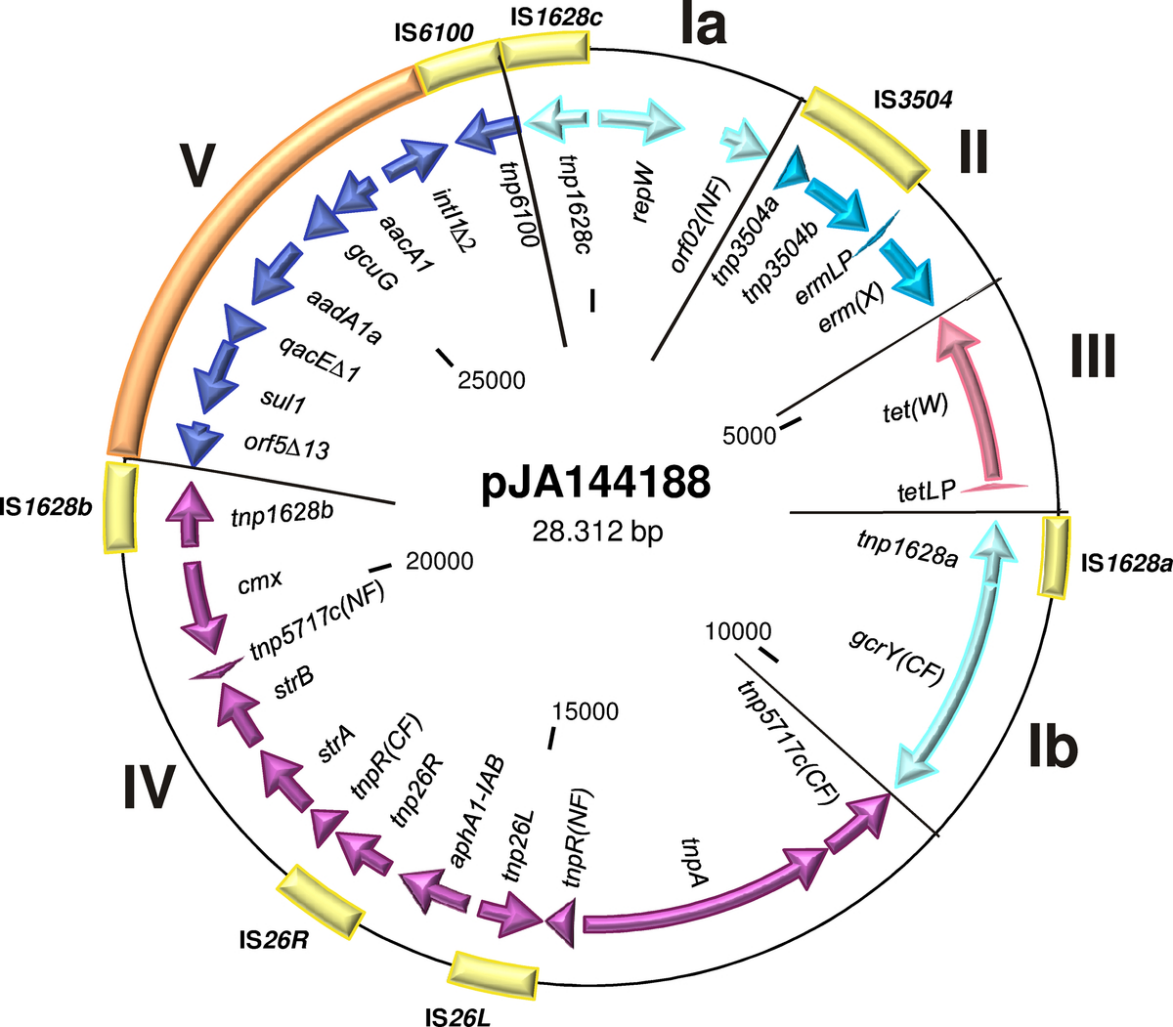
**Genetic map of the resistance plasmid pJA144188 from *C. resistens *DSM 45100**. The predicted protein-coding regions of pJA144188 are shown by arrows indicating the direction of transcription. The resistance plasmid can be divided into five distinct DNA modules (I to V) that are specifically colored. Insertion sequences (IS) are shown as yellow boxes. The position of a class 1 integron is marked by an orange box.

The genomic sequence of *C. resistens *DSM 45100 was determined by a whole-genome shotgun approach with pyrosequencing technology. A quarter of a single run with the Genome Sequencer FLX System and Titanium chemistry yielded 273,646 reads with a total number of 112,335,846 bases that were assembled into 73 large (≥ 500 bases) contigs and 19 small contigs. Bioinformatic analysis of the sequence assembly indicated that 14 contigs belong to a plasmid that was named pJA144188. The remaining gaps in the chromosome and in plasmid pJA144188 were closed by PCR strategies that were supported by the Consed program [16]. The final assemblies of the DNA sequences yielded a circular chromosome with a size of 2,601,311 bp (Figure 1A) and the 28,312-bp sequence of plasmid pJA144188 (Figure 2). Gene finding and annotation of the *C. resistens *DSM 45100 genome were performed with the GenDB software system [17] and resulted in the detection and characterization of 2,171 protein-coding regions on the chromosome. Furthermore, three *rrn *operons were detected with the RNAmmer tool [18], and 50 tRNA genes were predicted by the tRNAscan-SE program [19]. Relevant features deduced from the genome sequence of *C. resistens *DSM 45100 are summarized in Table 1. Plasmid pJA144188 consists of 31 protein-coding regions, of which nine were classified as pseudogenes. All pseudogenes of pJA144188 are remnants of protein-coding regions truncated by the transpositional integration of insertion sequences.

**Table 1:** Data deduced from the complete genome sequence of *C. resistens *DSM 45100

Feature	Chromosome	Plasmid pJA144188
Total size (bp)	2,601,311	28,312
G+C content (%)	57.1	55.3
No. of protein-coding sequences	2,171	31
Coding density (%)	87.9	87.4
Average gene length (bp)	1,053	798
No. of rRNAs	3 × 16S-23S-5S	0
No. of tRNAs	50	0
No. of CRISPRs^a^	73	0

^a ^Abbreviation: Clustered Regularly Interspaced Short Palindromic Repeats

### General architecture of the *C. resistens *DSM 45100 chromosome

The chromosome of *C. resistens *DSM 45100 revealed the typical features of a corynebacterial genome sequence. A plot of the calculated G/C skew [(G - C)/(G + C)] indicated a bi-directional replication mechanism of the *C. resistens *chromosome (Figure 1A). According to the presence and distribution of conserved DnaA boxes, the origin of replication (*oriC*) is located downstream of the *dnaA *coding region [20]. The biased distribution of architecture imparting sequences (AIMS) on the leading and lagging strands of the chromosome indicated the presence of a *dif *region involved in replication termination [21] at 1,233 kb on the chromosomal map, dividing the chromosome of *C. resistens *DSM 45100 into two replichores of nearly similar sizes (Figure 1B). A comparative analysis by reciprocal best matches with BLASTP [22] revealed a highly conserved order of orthologous genes between the chromosomes of *C. resistens *DSM 45100 and *C. jeikeium *K411 (Figure 1C). Since corynebacteria lack the *recBCD *recombination pathway [1,23], genetic rearrangements are generally rare in the respective genomes, although a moderate reorganization of the chromosomal architecture has been detected in species of the cluster 3 subline of the genus *Corynebacterium *[24-26]. The chromosomal synteny between *C. resistens *DSM 45100 and *C. jeikeium *K411 is interrupted due to a translocation of a 154-kb DNA region and the inversion of two distinct genomic segments in *C. resistens *(Figure 1C). As these inversions are part of the right replichore and as intra-replichore inversions are relatively rare [27], we assume that the current chromosomal architecture of *C. resistens *DSM 45100 resulted from a flip-flop of two consecutive inversions. Flip-flop means in this genomic context that the 125-kb central region of an initially inverted 270-kb DNA segment was probably inverted again to maintain the architectural bias in this part of the *C. resistens *chromosome (Figure 1B).

We therefore examined the gene-strand bias in the chromosome of *C. resistens *DSM 45100, taking into account that gene essentiality is a proposed driving force for the genetic organization in bacterial genomes [28]. In total, 58.7% of the protein-coding regions of *C. resistens *DSM 45100 are located on the leading strands of the chromosome, revealing a moderate gene-strand bias in this species. A comparative content analysis of predicted protein-coding regions from *C. resistens *DSM 45100 with candidate essential genes detected in the genome of *C. glutamicum *R by high-density transposon mutagenesis [29] revealed 365 candidate essential genes from *C. glutamicum *R having orthologs in the chromosome of *C. resistens*. The majority of these genes (75.1%) are located on the leading strands of the *C. resistens *chromosome, with 68.8% of all candidate essential genes being located on the left replichore, clearly indicating the prominent role of gene essentiality in bacterial gene-strand bias [28]. In the inverted genomic segment of the *C. resistens *chromosome, 46 candidate essential genes are located on the leading strand, whereas 43 candidate essential genes were detected on the lagging strand. This equal distribution of candidate essential genes on the leading and lagging strands might explain why an intra-replichore inversion has been established in the chromosome of *C. resistens *DSM 45100. It suggests furthermore that the orientation of the respective genes has no remarkable impact on the fitness of *C. resistens *DSM 45100.

**Figure 3:**
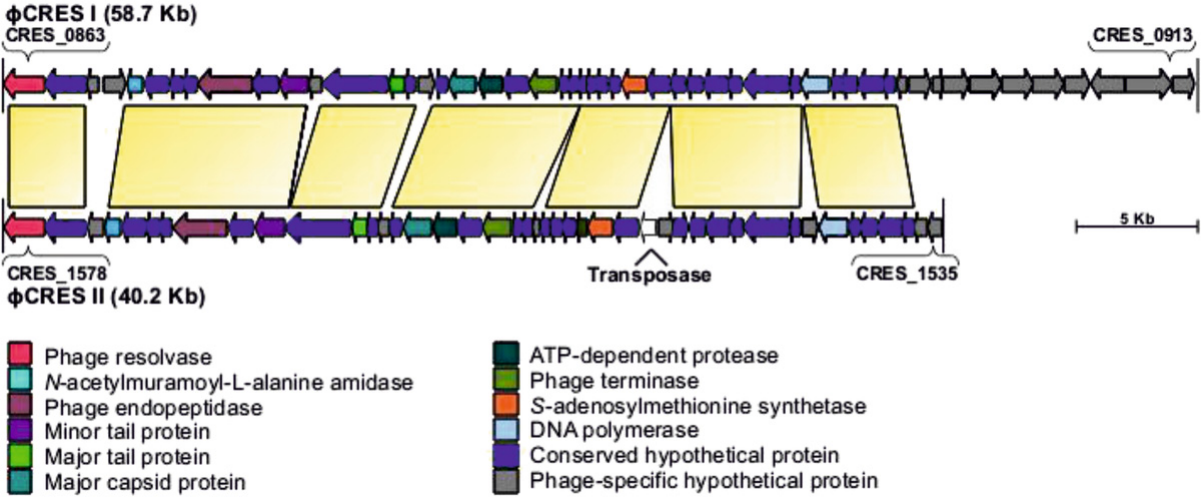
**Genetic organizations of prophages in *C. resistens *DSM 45100**. Arrows show genes and their direction of transcription. The color code indicates orthologs in both genomes and is explained in the figure. The integration of an insertion sequence into the genome of ΦCRES II is indicated by the coding region of the respective transposase.

Additional breakpoints of synteny between the chromosomes of *C. resistens *DSM 45100 and *C. jeikeium *K411 are caused by the presence of genes related to two prophages, named ϕCRES I and ϕCRES II (Figure 1C). The genomic segment of *C. resistens *DSM 45100 assigned to ϕCRES I has a size of about 58.7 kb and comprises 51 genes, whereas the ϕCRES II region has a size of about 40.2 kb and comprises 44 genes, including a transposase gene of an integrated insertion sequence (Figure 3). Both putative prophage genomes share not only a very similar set of protein-coding regions, but also a highly similar order of these genes, suggesting that the respective phages are genetically related (Figure 3). Moreover, a DNA region with 73 clustered regularly interspaced short palindromic repeats (CRISPRs) was detected in the chromosome of *C. resistens *DSM 45100 with the CRISPRFinder tool [30]. The CRISPR locus comprises DNA repeats with a length of 28 bp, which are separated by variable 33-bp spacer sequences. The adjacent genomic region in the chromosome of *C. resistens *DSM 45100 comprises seven CRISPR-associated genes, named *casA*-*casG*. The combination of highly similar CRISPRs and associated *cas *genes was detected previously in *C. jeikeium *K411 [25], *C. urealyticum *DSM 7109 [24], and *Nocardia farcinica *IFM 10152 [31] and can probably provide acquired resistance to bacteriophages [32].

A comparative content analysis of the predicted proteome of *C. resistens *DSM 45100 with the complete set of proteins encoded in the genomes of *C. jeikeium *K411 [25], *C. urealyticum *DSM 7109 [24], and *Corynebacterium kroppenstedtii *DSM 44385 [26] revealed that the four species belonging to the corynebacterial cluster 3 share 894 orthologous proteins, representing 41.2% of the predicted proteins from *C. resistens *DSM 45100 (data not shown). According to this comparative data, *C. resistens *DSM 45100 contains 563 species-specific genes that probably contribute to the characteristic phenotypic features of this bacterium. In the following sections, we analyze the gene repertoire of *C. resistens *DSM 45100 in more detail and deduce relevant features regarding the lipophilic lifestyle and the functions involved in virulence and multi-drug resistance of this new human pathogen.

### General metabolic features and lipophilic lifestyle of *C. resistens *DSM 45100

A bioinformatic reconstruction of the central carbon metabolism of *C. resistens *DSM 45100 revealed the absence of genes coding for the components of phosphoenolpyruvate:carbohydrate phosphotransferase systems (PTSs) (Additional file 1). The absence of the respective genes in *C. resistens *DSM 45100 was supported by TBLASTN searches with reference proteins from other corynebacteria or actinobacteria. PTSs play a major role in uptake and phosphorylation of numerous carbohydrates, as well as in monitoring the bacterial environment to choose alternative carbon sources for growth [33]. Only the *sugABCD *gene cluster encoding a putative sugar transport system of the ABC superfamily was detected in the genome of *C. resistens *DSM 45100 (Additional file 1). Moreover, the *glk *gene encoding glucokinase (EC 2.7.1.2.) and the *rbsK *gene coding for ribokinase (EC 2.7.1.15) are present in *C. resistens *DSM 45100, allowing the conversion of "free" sugars into phosphorylated central pathway intermediates. In accordance with this data, the taxonomic description of *C. resistens *indicated that glucose and ribose are catabolized by this species [14]. The *rbsK *gene is also part of a utilization pathway for the nucleoside uridine [34] that is imported into *C. resistens *by the major facilitator superfamily transporter UriT and converted to ribose and uracil by an inosine-uridine preferring nucleoside hydrolase (EC 3.2.2.1) encoded by the *uriH *gene (Additional file 1). Further metabolic analysis of the genome sequence revealed the presence of a complete set of genes involved in glycolysis, gluconeogenesis, and the pentose phosphate pathway. Likewise, the tricarboxylic acid cycle of *C. resistens *DSM 45100 and the glyoxylate bypass, comprising the genes *aceA *and *aceB*, are complete (Additional file 1). This is remarkable as the *sucCD *genes encoding subunits of succinyl-CoA synthetase (EC 6.2.1.5) are lacking in other sequenced genomes of cluster 3 corynebacteria [24-26]. On the other hand, enzymes catalyzing typical anaplerotic reactions in corynebacterial metabolism are not encoded in *C. resistens *DSM 45100, including phosphoenolpyruvate carboxylase (EC 4.1.1.31) and pyruvate carboxylase (EC 6.4.1.1). This observation suggests that *C. resistens *DSM 45100 is dependent on substrates for growth that are associated with the complete gluconeogenesis pathway.

The most likely substrates for growth of *C. resistens *are external fatty acids. *C. resistens *is often assigned to the group of "lipophilic" corynebacteria, whose growth is markedly enhanced by the addition of lipids to the culture medium [3]. This characteristic phenotype *per se *is a fatty acid auxotrophy that obviously originates from the lack of a fatty acid synthase gene (*fas*), which is generally responsible for the biosynthesis of fatty acids [35]. To satisfy the essential nutritional requirement for fatty acids as carbon and energy sources, a complete β-oxidation pathway is encoded in the genome of *C. resistens *DSM 45100 (Additional file 1). Ten *fadD *genes encoding acyl-CoA synthetases were identified in the genome of *C. resistens *DSM 45100, including the *fadD1 *gene that is involved in mycolic acid biosynthesis [36]. The *fadD10 *coding region represents a pseudogene as it is disrupted by an insertion sequence. Fatty acyl-CoA synthetases are generally involved in activating free fatty acids to form acyl-CoA of various chain lengths concomitant with the transport into the bacterial cell [37] and are also required for the utilization of endogeneous fatty acids released from membrane lipids [38]. The presence of a large number of orthologs and the amino acid sequence diversity of the fatty acyl-CoA synthetases of *C. resistens *DSM 45100 might indicate different substrate specificities of these enzymes. Other enzymes involved in the β-oxidation pathway of *C. resistens *DSM 45100 are encoded by seven paralogs of *fadE *(encoding acyl-CoA dehydrogenase), the bifunctional *fadB1 *gene (enoyl-CoA hydratase/hydroxyacyl-CoA dehydrogenase), the monofunctional *fadB2 *gene (hydroxyacyl-CoA dehydrogenase), five paralogs of *echA *(enoyl-CoA hydratase), and three paralogs of *fadA *(ketoacyl-CoA thiolase). The predicted amino acid sequences of the paralogous proteins vary substantially in *C. resistens *DSM 45100, again suggesting diverse substrate specificities of the respective enzymes. Moreover, the *acx *gene of *C. resistens *DSM 45100 encodes acyl-CoA oxidase (EC 1.3.3.6), which catalyzes the desaturation of fatty acyl-CoA thioesters and donates electrons directly to molecular oxygen, thereby producing H_2_O_2 _[39]. The subsequent detoxification of the resulting H_2_O_2 _is catalyzed by catalase (EC 1.11.1.6) encoded by the *katA *gene of *C. resistens *DSM 45100.

The degradation of modified fatty acyl-CoA esters requires the recruitment of auxiliary enzymes to link their utilization to the main β-oxidation pathway [39]. The *fadH *gene for instance encodes 2,4-dienoyl-CoA reductase (EC 1.3.1.34), which is required for the degradation of unsaturated fatty acids, whose double bond extends from an even-numbered carbon atom. Moreover, the genes *prpC *and *prpD *are involved in the metabolism of propionyl-CoA via the 2-methylcitrate cycle [40]. Propionyl-CoA can result from β-oxidation of odd-chain fatty acids and is converted to 2-methylisocitrate by the consecutive reactions of 2-methylcitrate synthase (EC 2.3.3.5) encoded by the *prpC *gene, and 2-methylcitrate dehydratase (EC 4.2.1.79) encoced by *prpD*. The last step of this cycle, the cleavage of 2-methylisocitrate to succinate and pyruvate, is catalyzed by 2-methylisocitrate lyase (EC 4.1.3.30) that is not encoded in the genome of *C. resistens *DSM 45100. Despite the lack of a corresponding *prpB *gene to complete the 2-methylcitrate cycle, the oxidation of odd-chain fatty acids by *C. resistens *DSM 45100 seems possible when considering that isocitrate lyase (AceA) might also function as 2-methylisocitrate lyase, as it was demonstrated in *Mycobacterium tuberculosis *[40]. *C. resistens *DSM 45100 can also channel propionate into the tricarboxylic acid cycle via the enzymatic reactions encoded by the methylcitrate cycle genes (Additional file 1). Propionate is imported into *C. resistens *DSM 45100 by a monocarboxylic acid transporter encoded by the *mctC *gene [41].

The activation of fatty acids to acyl-CoA thioesters is not only the initial step of the β-oxidation pathway, but also for the biosynthesis of corynomycolic acids [42]. Mycolic acids are major constituents of the corynebacterial cell envelope and synthesized by the polyketide synthase Pks13 [42] and the reductase CmrA [43]. The *pks13 *coding region of *C. resistens *DSM 45100 is located in a conserved gene cluster [44], including genes coding for an acyl-CoA carboxylase (*accD3*), an acyl-CoA synthetase/acyl-AMP ligase (*fadD1*), the envelope lipids regulation factor ElrF (*elrF*), and two trehalose corynomycol transferases (*cmtB *and *cmtC*). A third gene coding for a corynomycolyl transferase (*cmtA*) is located elsewhere in the chromosome of *C. resistens *DSM 45100. Trehalose corynomycol transferases catalyze the transfer of mycolic acids from trehalose monocorynomycolate on the cell wall arabinogalactan and on another trehalose monocorynomycolate to yield trehalose dicorynomycolate [45].

Another gene cluster involved in fatty acid metabolism of *C. resistens *DSM 45100 includes genes coding for α and β subunits of acyl-CoA carboxylase (*accD1 *and *accBC1*), an acyl-CoA dehydrogenase (*fadE8*), a putative enoyl-CoA hydratase domain-containing protein (*echC*), a citrate lyase β-subunit (*citE*), an acyl-CoA synthetase (*fadD5*), and a ketoacyl-CoA thiolase (*fadA3*). The regulatory gene *tetR *encoding a regulator of the TetR protein family is located in front of the *accD1 *gene and might be involved in the transcriptional control of the complete gene cluster. A similar arrangement of genes is present only in the genomes of the lipophilic species *C. jeikeium *K411 and *Corynebacterium amycolatum *SK46, whereas a subset of genes (including a regulatory *tetR *gene) was found also in the genome of *M. tuberculosis *H37Rv [46]. As most of the proteins encoded in these conserved gene clusters are linked to fatty acid catabolism, they might be involved in the activation and subsequent degradation of a hitherto unknown fatty acid substrate.

### Amino acid metabolism and utilization of histidine by *C. resistens *DSM 45100

**Figure 4:**
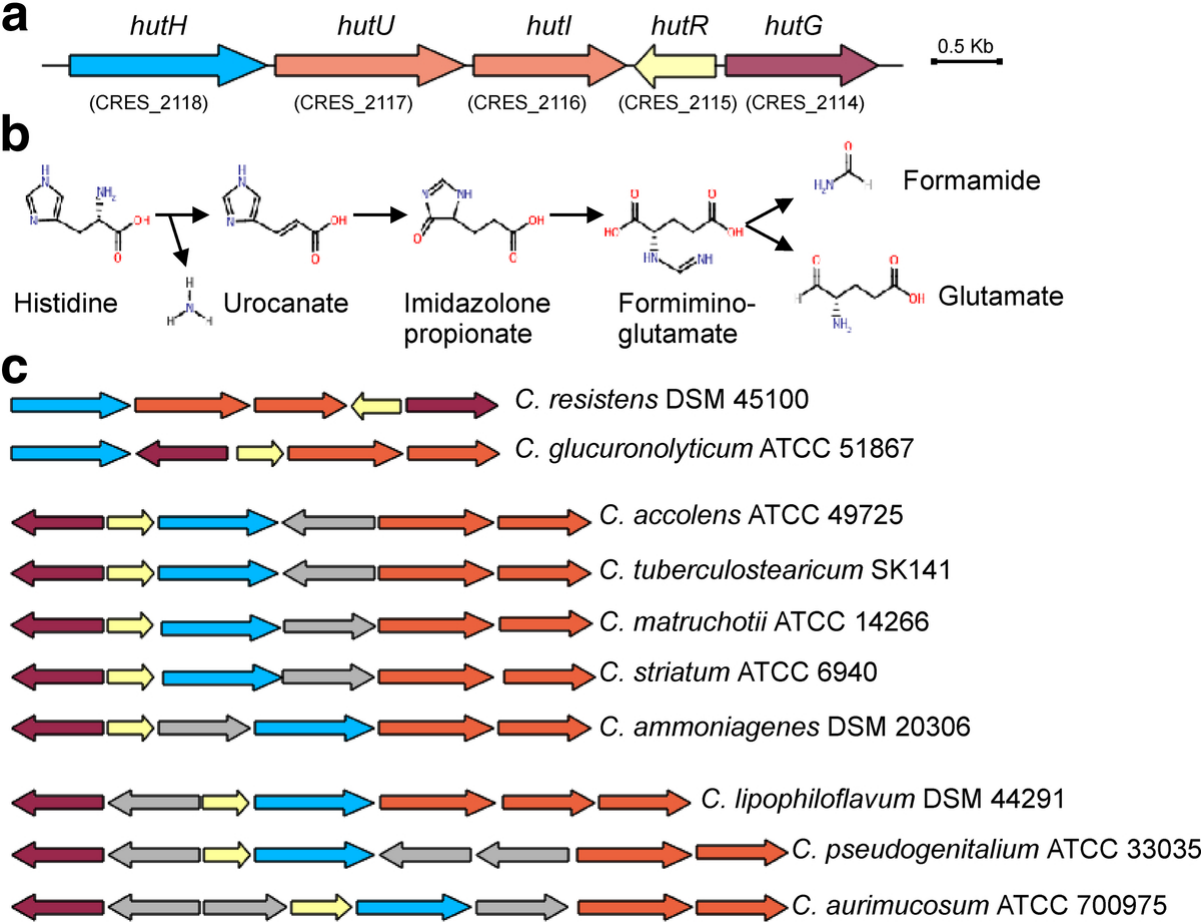
**Genetic organizations of *hut *gene regions in *C. resistens *DSM 45100 and other corynebacteria**. **(A)**, Map of the *hut *gene region of *C. resistens *DSM 45100. Arrows represent *hut *genes and indicate their direction of transcription. The *hut *genes are labeled with names and identifiers. **(B)**, Metabolic pathway for the catabolism of L-histidine. Enzymes of the four-step *hut *pathway are: HutH, histidine ammonia-lyase; HutU, urocanate hydratase; HutI, imidazolonepropionase; HutG, formimidoylglutamase; HutR, transcriptional regulator of the IclR superfamily. **(C)**, Comparative analysis of *hut *gene regions in corynebacterial species. Genes with similar color participate in the same enzymatic step of the pathway. Additional genes present in the respective genomic regions are shown in grey.

According to the genome annotation, all currently known pathways for the biosynthesis of standard proteinogenic amino acids are present in *C. resistens *DSM 45100 (Additional file 2). The genome sequence of *C. resistens *DSM 45100 contains moreover the *agxT *gene encoding serine-glyoxylate aminotransferase (EC 2.6.1.45) that catalyzes the conversion of L-serine and glyoxylate to 3-hydroxypyruvate and glycine, the *sdaA *gene encoding L-serine dehydratase (EC 4.3.1.17) involved in the conversion of L-serine to pyruvate and NH_3_, and the *arcB *gene encoding ornithine cyclodeaminase (EC 4.3.1.12) that converts L-ornithine to L-proline and NH_3 _(Additional file 2). Another enzymatic reaction that generates NH_3 _is carried out by histidine ammonia-lyase (HutH; EC 4.3.1.3). This enzyme catalyzes the first step in the degradation of L-histidine and the product, urocanate, is further metabolized to glutamate and formamid [47]. A complete histidine utilization (*hut*) pathway was identified in *C. resistens *DSM 45100 and is represented by the *hut *gene cluster (Figure 4A). The products of this gene cluster catalyze the four-step conversion of L-histidine to L-glutamate (Figure 4B). The first enzymatic reaction of this pathway is catalyzed by HutH, followed by the conversion of the resulting urocanate to 4-imidazolone propanoate by urocanate hydratase (HutU; EC 4.2.1.49). Formiminoglutamate is generated in the third step by imidazolonepropionase (HutI; EC 3.5.2.7) and is finally hydrolyzed into L-glutamate and formamide by formimidoylglutamase (HutG; EC 3.5.3.8).

A comparative analysis of *hut *gene regions detected in the genus *Corynebacterium *revealed different genetic organizations of the respective clusters in ten corynebacterial species, with *C. resistens *DSM 45100 representing a new order of *hut *genes (Figure 4C). All *hut *gene clusters contain the *hutR *gene, which encodes a transcription regulator of the IclR protein family that is probably involved in the transcriptional control of histidine utilization in corynebacteria. It is remarkable that the majority of corynebacterial species harboring a *hut *gene cluster are in some way associated with the urogenital tract. *Corynebacterium glucuronolyticum, Corynebacterium tuberculostearicum*, and *Corynebacterium pseudogenitalium *were isolated from the urogenital tract of males and females [48-50], whereas *Corynebacterium lipophiloflavum *was isolated from bacterial vaginosis [51]. Black-pigmented *Corynebacterium aurimucosum *isolates derive from vaginal swabs and probably cause spontaneous abortion [52]. Indeed, variable amounts of L-histidine are present in the human vaginal fluid [53] and might be used by these bacteria as a combined nitrogen and carbon source for growth. *C. resistens *DSM 45100 might also use L-histidine as a carbon and/or nitrogen source, thus compensating for the restricted availability of carbohydrates due to the strict lipophilic lifestyle. The natural habitat of *C. resistens *is currently unknown, although the utilization of L-histidine by the enzymatic machinery of the *hut *pathway points to a colonization of the human inguinal or perineal regions, thereby living in close proximity to the human genital tract. This hypothesis is consistent with previous microbiological studies that recovered lipophilic corynebacteria predominantly from the axillary, inguinal, and perineal areas of the human body [54]. These sites of the human body are characterized by an elevated moisture of the skin in conjunction with a substantial formation of hydrolipid films, which are composed of triacylglycerides, free fatty acids, ceramides, cholesterol, and cholesterol esters. These compounds are appropriate carbohydrate substrates for the growth of lipophilic corynebacteria. Additional experimental evidence to support the hypothesis that *C. resistens *is a colonizer of the inguinal and/or perineal areas of the human body is currently lacking, as no 16S rDNA sequences of *C. resistens *were detected in the course of the human microbiome project already covering several body sites, including the human urogenital tract [55].

### Detection of candidate virulence factors in the genome of *C. resistens *DSM 45100

To better understand the pathogenic potential of *C. resistens *DSM 45100, the genome sequence was furthermore screened for genes encoding candidate virulence factors, which in principle should be part of the exoproteome of this species. To estimate the number of secreted proteins encoded by *C. resistens *DSM 45100, the first 70 amino acid residues of each predicted protein were used to search for amino-terminal signal peptides with SignalP 4.0 [56]. In this way, a total number of 254 proteins were identified to be potentially secreted by *C. resistens *DSM 45100. However, it has to be considered that proteins with signal peptides might be destined for the integration into the cytoplasmic membrane and therefore contain membrane-spanning domains [57]. A total number of 258 predicted proteins with membrane-spanning domains were detected by the TMHMM tool [58], and the combined feature of signal peptides and membrane-spanning domains was found in a subset of 78 proteins of *C. resistens *DSM 45100. The remaining proteins were screened for predicted functions probably related to the virulence of *C. resistens *DSM 45100 (Table 2).

**Table 2:** Candidate virulence factors detected in the chromosome of *C. resistens *DSM 45100

Identifier	Gene	Predicted protein function (and putative role in virulence)
CRES_2101	*surA*	surface protein (cell surface variation)
CRES_0606	*surB*	surface protein (cell surface variation)
CRES_0405	*spaA*	major pilin subunit of the SpaABC pilus (adhesion)
CRES_0407	*spaB*	minor pilin subunit of the SpaABC pilus (adhesion)
CRES_0408	*spaC*	tip protein of the SpaABC pilus (adhesion)
CRES_1049	*rpfI*	resuscitation-promoting factor-interacting protein (adhesion)
CRES_0767	*cwlH*	cell wall-associated hydrolase (adhesion)
CRES_0700	*choD*	cholesterol oxidase (oxidation of cholesterol)
CRES_1191	*asa*	alkaline ceramidase (hydrolysis of ceramides)
CRES_0207	*lipS1*	lipase of the LIP superfamily (lipolytic activity)
CRES_1004	*lipS2*	lipase of the LIP superfamily (lipolytic activity)
CRES_2090	*lipS3*	lipase of the LIP superfamily (lipolytic activity)
CRES_0539	*sgnH*	esterase of the SGNH-hydrolase superfamily (lipolytic activity)

*C. resistens *DSM 45100 encodes two cell surface protein precursors, named SurA and SurB, which contain carboxyterminal sorting (LPxTG) signals recognized by sortase transpeptidase. The housekeeping sortase of *C. resistens *DSM 45100 (SrtC) is most likely responsible for anchoring these LPxTG-containing proteins to the corynebacterial cell wall [59]. The carboxyterminal part of the SurB protein contains a remarkable tandem repeat region with the consensus sequence PGTTTPGTTA that is present 13 times with only moderate variations in the amino acid sequence. Additional repeat regions with the consensus sequences WATVNPDGS or VVVTYPDGS are present in the central region of the cell surface protein. The SurB protein of *C. resistens *DSM 45100 is thus structurally similar to the alpha C protein-antigen of group B streptococci containing large tandem repeating units [60]. Variations of the number of tandem repeat regions of the alpha C protein affected the pathogenicity of group B streptococci [61], and the structural variations of the bacterial cell surface conferred protective immunity against the host defense [62].

Another structural component of the cell surface of *C. resistens *DSM 45100 is an adhesive pilus of the SpaABC type (Table 2). Cell-surface pili are important virulence factors that enable pathogens to adhere to specific host tissues and to modulate host immune response [63]. The SpaABC pilus of *C. resistens *DSM 45100 is covalently anchored to the corynebacterial cell wall by the pilin-specific sortases SrtA and SrtB via a transpeptidylation mechanism [59]. The adhesive pilus of *C. resistens *DSM 45100 consists of three pilin subunits encoded by the *spaABC *genes. The *spaA *gene encodes the major pilin of the pilus shaft, whereas the *spaB *and *spaC *genes code for minor pilins located at the base and at the tip of the pilus, respectively. The homologous pilus structure of *C. diphtheriae *NCTC 13129 can mediate the adhesion of the pathogen to human pharyngeal epithelial cells, which is a crucial step during infection [64].

Further candidate virulence factors that may support the adhesion of *C. resistens *DSM 45100 to host cells are encoded by the *rpfI *and *cwlH *genes (Table 2). The deduced proteins revealed amino acid sequence homology to DIP1281 and DIP1621 from *C. diphtheriae *NCTC 13129, respectively. The *rpfI *gene encodes a resuscitation-promoting factor-interacting protein that forms complexes with lytic transglycosylases (resuscitation-promoting factors) at the septum of dividing bacteria [65]. *C. resistens *DSM 45100 encodes two resuscitation-promoting factors, named RpfA and RpfB, which may interact with the RpfI protein. The homologous gene product DIP1281 was shown to be crucial for adhesion and colonization of host epithelial cells [66]. Defined DIP1281 mutant cells of *C. diphtheriae *completely lacked the ability to adhere to host cells and to invade these [66]. Due to the interaction of RpfI with resuscitation-promoting factors, it is probably involved in the organization of the outer surface layer of the pathogen and might thereby impair the efficiency of adhesion. The *cwlH *gene of *C. resistens *DSM 45100 encodes a cell wall-associated hydrolase with a carboxyterminal domain similar to proteins belonging to the NlpC/P60 family [67]. The targeted disruption of the homologous DIP1621 gene in *C. diphtheriae *led to decreased adherence to epithelial cells; but the exact function of this protein remains unknown so far [68].

Among the candidate virulence factors detected in *C. resistens *DSM 45100 is also a secreted cholesterol oxidase encoded by the *choD *gene (Table 2). The deduced ChoD protein is a putative membrane-damaging toxin, probably causing the enzymatic oxidation of macrophage membrane cholesterol [69]. Cholesterol oxidase is an important cytolytic factor for *Rhodococcus equi *as its presence was accompanied by intracellular survival of this pathogen, whereas a non-virulent strain lacking this enzyme was largely eliminated from the macrophages [69]. Likewise, a *choD *mutant of *M. tuberculosis *was attenuated in peritoneal macrophages, whereas no attenuation was observed when the same strain was complemented with an intact *choD *gene [70]. The oxidation of membrane cholesterol might lead to total disorganization of the eukaryotic cell membrane [71], supporting the release of substrates for other enzymes involved in fatty acid metabolism of a pathogen. Another enzyme representig a candidate virulence factor of *C. resistens *DSM 45100 is the secreted alkaline ceramidase encoded by the *asa *gene (Table 2). Ceramidases hydrolyze the amide bond in ceramides, which results in the release of free fatty acids and sphingosine [72]. Sphingolipids are components of eukaryotic cell membranes, and hence they are putative targets for acquiring fatty acids by means of eukaryotic membrane damage. Moreover, the release of sphingosine by alkaline ceramidases is known for instance to attenuate the activity of macrophages [73].

*C. resistens *DSM 45100 can also generate free fatty acids from the host tissue by secreting lipolytic enzymes (Table 2). Three secreted lipases of the LIP superfamily containing enzymes with broad lipolytic activities are encoded in the genome of *C. resistens *DSM 45100 by *lipS1, lipS2*, and *lipS3*. These enzymes may thus contribute to the generation of free fatty acids from precursor molecules such as triacylglycerol. The prototype enzymes of the LIP superfamily were studied in *Candida albicans*, where these lipases are expressed and secreted during the infection cycle of this pathogen and may contribute to the persistence and virulence of *C. albicans *in human tissue [74]. The *sgnH *gene of *C. resistens *DSM 45100 was also classified as a candidate virulence factor (Table 2). It encodes a secreted hydrolase of the SGNH superfamily, which is a group of enzymes that hydrolyze ester bonds in lipids [75]. SGNH enzymes have little sequence homology to other lipases and are characterized by the four invariant catalytic residues serine, glycine, asparagine, and histidine. Due to a flexible active site that appears to change conformation with the presence of different substrates, SGNH esterases and lipases are hydrolytic enzymes with multifunctional properties, such as broad substrate specificities [75]. In summary, numerous candidate virulence factors of *C. resistens *DSM 45100 are obviously linked to the strict lipophilic lifestyle of this species by providing essential nutrients for bacterial growth.

### The penicillin-binding proteins and the quinolone-resistance-determining region of *C. resistens *DSM 45100

In addition to lipophilism and virulence, multi-drug resistance is another prominent feature of the hitherto detected clinical isolates of *C. resistens *[14]. The relevance of chromosomal genes for the multi-drug resistance profile of *C. resistens *DSM 45100 is apparent when considering the results of the initial antimicrobial susceptibility assays with several β-lactams, the most broadly used class of antimicrobials, and the fluoroquinolone antibiotic ciprofloxacin. All *C. resistens *isolates were characterized by high minimum inhibitory concentrations (MICs) of the selected antibiotics [14]. The resistance of *C. resistens *DSM 45100 to β-lactams might be associated with the presence of antibiotic-insensitive types of penicillin-binding proteins [76]. The chromosome of *C. resistens *DSM 45100 encodes six penicillin-binding proteins (PBPs) belonging to three protein families. PBP1A and PBP1B are bifunctional transglycosylases/transpeptidases of the high-molecular weight PBP 1A family, whereas the proteins PBP2A, PBP2B, and PBP2C act as transpeptidases and are members of the high-molecular weight PBP 2 family [67]. The Dac protein of *C. resistens *DSM 45100 represents a D-alanyl-D-alanine carboxypeptidase of the low-molecular weight PBP 4 family [67]. Moreover, *C. resistens *DSM 45100 contains two genes, *ldt1 *(CRES_0602) and *ldt2 *(CRES_0140), encoding putative L,D-transpeptidases. These enzymes can act in an alternative pathway for peptidoglycan cross-linking and can thus contribute to the resistance to β-lactam antibiotics that inhibit the penicillin-binding proteins, which usually catalyze the cross-linking reaction [67]. In *C. jeikeium *K411, the high-molecular weight penicillin-binding protein PBP2C and the L,D-transpeptidase Ldt1 were shown to be two ampicillin-insensitive cross-linking enzymes involved in peptidoglycan biosynthesis [77].

Resistance to fluoroquinolones is often caused by mutations in the so-called quinolone-resistance-determining region (QRDR) of the gyrase gene *gyrA *[78]. The minimum inhibitory concentrations of fluoroquinolones determined in this study revealed high-level resistances of *C. resistens *DSM 45100 to danofloxacin (32 μg ml^-1^), ciprofloxacin, levofloxacin, sparfloxacin (64 μg ml^-1^), and norfloxacin (128 μg ml^-1^). Single amino acid substitutions in position 90 of the GyrA protein (*C. resistens *numbering) are generally sufficient to generate fluoroquinolone resistance in corynebacteria, but double mutations in the *gyrA *gene leading to changes in positions 90 and 94 of the gene product are necessary for high-level resistances [79]. The GyrA protein of *C. resistens *DSM 45100 contains typical amino acid residues in the deduced QRDR that are related to high-level fluoroquinolone resistance. In particluar, the amino acid sequence motif LAIYG is characterized by the Leu-90 and Gly-94 residues, which were already associated with high-level resistances to ciprofloxacin, levofloxacin, and norfloxacin in clinical isolates of *Corynebacterium macginleyi *[79,80]. Likewise, specific double mutations in the QRDR of the *gyrA *genes from *Corynebacterium striatum *and *Corynebacterium amycolatum *resulted in amino acid changes in positions 90 and 94 of the GyrA proteins (*C. resistens *numbering) and in high levels of fluoroquinolone resistance [79,80]. Moreover, single mutations in the *gyrA *gene of *Escherichia coli *leading to changes of the deduced QRDR sequence SAVYD to either LAVYD or SAVYG were associated with resistances to ciprofloxacin and ofloxacin [81]. It is thus very likely that specific mutations in the QRDR of the *gyrA *gene of *C. resistens *DSM 45100 are responsible for high-level resistances to fluoroquinolones. Other antibiotic resistance phenotypes of *C. resistens *DSM 45100 are apparently associated with the presence of plasmid pJA144188 that is analyzed in more detail in the following section.

### The modular architecture of the multi-drug resistance plasmid pJA144188

The annotation of the complete nucleotide sequence of plasmid pJA144188 from *C. resistens *DSM 45100 revealed a modular genetic structure of this replicon (Figure 2). The backbone of the plasmid is apparently loaded with several mobile genetic elements and antibiotic resistance genes, including a new class 1 integron. The insertion sequences and transposons of pJA144188 form the boundaries of five distinct DNA segments, each most probably acquired by horizontal gene transfer (Figure 2). The DNA segments of pJA144188 were assigned as follows: (module I) replication region and plasmid backbone with similarity to the multi-drug resistance plasmid pTP10 from the opportunistic human pathogen *C. striatum *M82B; (module II) macrolide-lincosamide-streptogramin (MLS) resistance region with similarity to pNG2 from the human pathogen *C. diphtheriae *S601; (module III) tetracycline resistance region with similarity to pLR581 from *Lactobacillus reuteri *ATCC 55730, which encodes the ribosomal protection protein Tet(W) and is reported here for the first time to occur in corynebacteria; (module IV) chloramphenicol and aminoglycoside resistance region with similarity to the Tn*45 *family transposon Tn*5717a *from the human pathogen *C. urealyticum *DSM 7109; (module V) class 1 integron that is specified by the presence of the rare *aacA1*:*gcuG *gene pair and the *aadA1a *gene cassette.

The small plasmid backbone of pJA144188 (module I) is characterized by the presence of the *repW *gene encoding the replication initiator protein RepW, whose amino acid sequence contains the characteristic signature motif GVPYGKYPR of IncW plasmids [82] and is almost identical to the RepA protein of pTP10 from *C. striatum *M82B [83]. Plasmid pJA144188 is thus a new member of the small IncW family of corynebacterial plasmids that probably uses the theta-type mechanism for replication [82]. The IncW family of corynebacterial plasmids includes moreover the bacteriocin-producing plasmid pKW4 from *C. jeikeium *K411 [25], the cryptic plasmid pCRY4 from *C. glutamicum *LP-6 obtained from a pig-manure deodorizing plant [84] and the low-copy-number plasmid pLEW279b from *Corynebacterium *sp. L2-79-05 isolated from poultry litter [85]. Characteristic 22-bp iterons, previously detected also on plasmid pTP10 [83], are present downstream of the *repW *coding region on pJA144188, occurring seven times. Such multiple sites of directly repeated sequences were identified in the origin regions of several plasmids. They are essential DNA-binding sites of the plasmid-specific replication initiator protein and have additional replication and copy number control properties [86]. As the remaining genes of pJA144188 are not related to typical plasmid replication and maintenance functions, it is most likely that the *repW *gene region and the replication initiator protein RepW are solely responsible for the stable inheritance of pJA144188 in *C. resistens *DSM 45100.

### DNA modules of plasmid pJA144188 containing antibiotic resistance regions

Module II of plasmid pJA144188 includes the *erm*(X) gene encoding a 23S rRNA adenine *N*-6-methyltransferase [87]. The *erm*(X) gene is preceded by IS*3504 *and a short leader peptide gene that might be involved in posttranscriptional regulation of *erm*(X) expression by erythromycin-inducible translational attenuation [88]. An almost identical DNA region is present on plasmid pNG2 from the erythromycin-resistant human pathogen *C. diphtheriae *S601 [89] that was isolated during an outbreak of diphtheria in Seattle [90]. Previous antimicrobial susceptibility assays demonstrated that the *erm*(X) gene provides high resistance levels to clinically relevant macrolides and lincosamides, such as erythromycin, azithromycin, josamycin, midecamycin, roxithromycin, spiramycin, tylosin, clindamycin, and lincomycin, and to the streptogramin B antibiotics quinupristin and pristinamycin I_A _[83,91,92]. This tremendous cross-resistance profile of Erm(X) can be understood when considering the common binding site of MLS antibiotics in the bacterial ribosome that is determined by the A2058 residue (*E. coli *numbering) in the large ribosomal subunit RNA [93].

Module IV of plasmid pJA144188 comprises the complex structure of transposon Tn*5717c *that is highly similar to transposon Tn*5717a *from the chromosome of *C. urealyticum *DSM 7109 (Figure 2). Tn*5717c *is thus an interlacing of the chlorampenicol resistance transposon Tn*45*, the streptomycin resistance transposon Tn*5393*, and the aminoglycoside resistance transposon Tn*5715*, and seems to have its seed in Tn*45 *detected on pXZ10145 from *C. glutamicum *1014 [94] and in the chromosome of *C. urealyticum *DSM 7109 [24]. Transposon Tn*45 *is an unusual mobile genetic element in corynebacteria that consists of a transposase gene and the *cmx *gene coding for a chlorampenicol efflux protein of the major facilitator superfamily [83]. Transposon Tn*5393 *is, on the other hand, a typical mobile genetic element of the Tn*3 *family and contains the *strA*-*strB *tandem pair of antibiotic resistance genes. The former gene encodes the aminoglycoside 3"-phosphotransferase APH(3")-Ib and the latter gene the aminoglycoside 6-phosphotransferase APH(6)-Id, both specifically conferring streptomycin resistance [95]. The association of the *strA*-*strB *genes with variants of transposon Tn*5393 *is also found in Gram-negative phytopathogenic bacteria, such as *Erwinia amylovora, Pseudomonas syringae*, and *Xanthomonas campestris*, where the Tn*5393 *elements occur on large conjugative plasmids [96]. The composite transposon Tn*5715 *harbors the aminoglycoside resistance gene *aphA1-IAB *encoding a member of the aminoglycoside 3'-phosphotransferase protein family, APH(3')-Ic [97]. The expression of the *aphA1-IAB *gene from the R-plasmid pTP10 in the susceptible host *C. glutamicum *ATCC 13032 revealed high-level resistances to kanamycin, neomycin, lividomycin, paromomycin, and ribostamycin [83] and thus the characteristic substrate profile of an APH(3')-I enzyme [98]. The *aphA1-IAB *gene present on pJA144188 may therefore confer resistance to a selected set of aminoglycoside antibiotics in *C. resistens *DSM 45100. However, a mininum inhibitory concentration of 16 μg ml^-1 ^was detected for the aminoglycoside amikacin in the initial taxonomic description of *C. resistens *DSM 45100 [14]. This observation indicates that additional resistance determinants are present in *C. resistens *DSM 45100 and confer a broader spectrum of aminoglycoside resistances.

### The class 1 integron of plasmid pJA144188 and its gene cassettes encoding aminoglycoside resistance proteins

**Figure 5:**
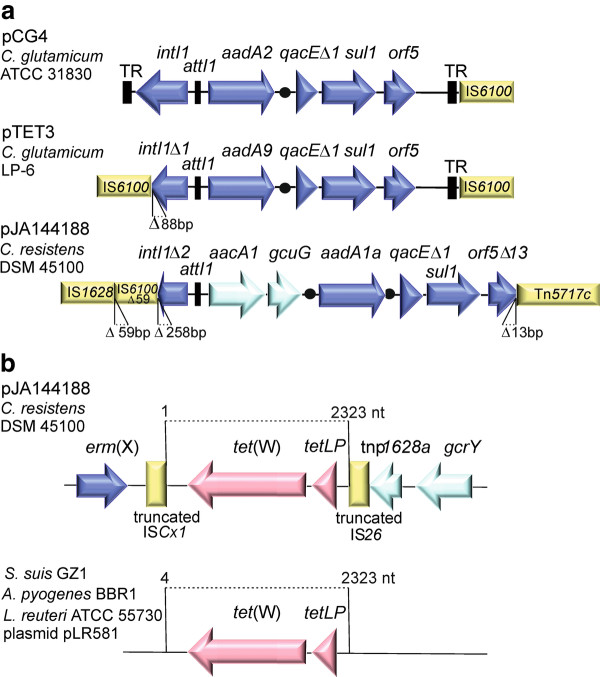
**Prominent genetic features of plasmid pJA144188 from *C. resistens *DSM 45100 (A), Genetic structure of corynebacterial class 1 integrons**. A comparison of the class 1 integron from pJA144188 with those present on plasmids pTET3 and pCG4 from *C. glutamicum *strains is shown. The filled circles indicate the position of 59-bp elements downstream of gene cassettes. The *aacA1 *and *gcuG *genes represent a gene pair that is not separated by a 59-bp element. The cassette integration site *attI1 *and the terminal repeat sequences (TR) are shown as black boxes. Deletions characterizing the class 1 integrons on pJA144188 and pTET3 are indicated. **(B)**, Detailed view of the tetracycline resistance gene region of pJA144188. The *tet*(W) gene region is presented including the predicted leader peptide gene *tetLP*. The truncated insertion sequences IS*Cx1 *and IS*26 *flanking the *tet*(W) gene region are shown as yellow boxes. These remnants of insertion sequences form the boundaries of a 2,323 bp DNA sequence that is also present in *Streptococcus suis, Arcanobacterium pyogenes*, and *Lactobacillus reuteri*, with the exception of three terminal nucleotides at the 5' end.

Module V of plasmid pJA144188 comprises typical genes of class 1 integrons that constitute genetic systems for gene capture and gene expression and are composed of conserved 5' and 3' segments [99,100]. The 5' conserved segment contains an integrase gene *intI1*, followed by the recombination site *attI1*, where gene cassettes are integrated by site-specific recombination after the integrase has recognized their 59-bp element. The 3' conserved segment of class 1 integrons is often specified by the presence of the *qacE*Δ*1, sul1*, and *orf5 *genes [99,100]. The *sul1 *gene encodes dihydropteroate synthase that can confer resistance to a broad spectrum of sulfonamides. The class 1 integron of pJA144188 is characterized by small deletions in the 5' and 3' conserved segments and by a gene cassette array that comprises three coding regions: the rare *aacA1*:*gcuG *tandem gene cassette and the *aadA1a *gene cassette (Figure 5A). The aminoglycoside resistance gene *aadA1a *encodes the aminoglycoside 3"-adenyltransferase ANT(3")-Ia with a specific substrate profile comprising only streptomycin and spectinomycin [98]. The *aacA1 *gene encodes the aminoglycoside 6'-acetyltransferase AAC(6')-Ia that can confer resistance to kanamycin, amikacin, dibekacin, netilmicin, sisomicin, and tobramycin [98]. It is thus likely that the *aacA1 *gene of pJA144188 mediates the observed resistance of *C. resistens *DSM 45100 to amikacin [14].

It is remarkable that the *aacA1 *resistance gene and the *gcuG *gene of unknown function represent a gene pair that is present in a single gene cassette and thus not separated by a 59-bp element [100]. The fused *aacA1*:*gcuG *gene cassette is rare in class 1 integrons and it has been observed in corynebacteria for the first time in the present study. Other class 1 integrons containing the *aacA1*:*gcuG *gene pair were detected, for instance, on plasmid pKGB525 from *Klebsiella pneumoniae *[101], on pCMXR1 from *E. coli *HKYM68 [102], and on the R factor NR79 from *E. coli *W677 [103]. Commonly, integrons are features of Gram-negative bacteria and only few integrons have been reported from Gram-positive bacteria, including two class 1 integrons from corynebacteria (Figure 5A). The first corynebacterial integron was detected on plasmid pCG4 from *C. glutamicum *ATCC 31830 [104] and the second element on plasmid pTET3 from *C. glutamicum *LP-6 [105]. The integron of pCG4 contains the *aadA2 *gene cassette, whereas the *aadA9 *gene cassette was detected on pTET3. Both genes confer streptomycin-spectinomycin resistance and encode aminoglycoside 3"-adenyltransferases of the ANT(3")-I protein family [98]. Accordingly, plasmid pJA144188 carries a new class 1 integron with two gene cassettes probably contributing to the extended spectrum of aminoglycoside resistances in *C. resistens *DSM 45100.

### The tetracycline-minocycline resistance region of plasmid pJA144188

Module III of plasmid pJA144188 contains the *tet*(W) gene, which is preceded by the putative leader peptide gene *tetLP *(Figure 2). The deduced Tet(W) protein revealed 99% identity, with only three substitutions in the amino acid sequence, to Tet(W) encoded on plasmid pLR581 from *Lactobacillus reuteri *ATCC 55730, a commercially available probiotic strain [106]. Tet(W) represents a ribosomal protection protein (RPP) that can promote high-level resistance to tetracyclines in Gram-positive and Gram-negative bacteria [107]. RPPs are supposed to originate from bacterial elongation factors and mediate tetracycline resistance by a complex molecular mechanism: They dislodge tetracycline from the ribosome, which is occupied by the antibiotic, such that an aminoacyl-tRNA can bind to the A site of the ribosome and protein biosynthesis can continue. RPPs can thus overcome the antimicrobial effect of typical tetracyclines, which bind to the ribosome and inhibit the elongation phase of protein biosynthesis [107]. The tetracycline resistance region of pJA144188 covers a 2,323-bp DNA sequence that is almost identical to *tet*(W) gene regions in *Streptococcus suis *GZ1 [108], *Arcanobacterium pyogenes *BBR1 [109], and to *tet*(W) on plasmid pLR581 from *L. reuteri *ATCC 55730 [106] (Figure 5B). This DNA segment obviously represents a conserved *tet*(W) core region in these Gram-positive species, whereas the flanking sequences of *tet*(W) genes are highly diverse in these species and in other bacteria [110]. The boundaries of the tetracycline resistance region on pJA144188 are clearly defined by the presence of two remnants of insertion sequences (Figure 5B). The truncated IS*Cx1 *element is known from the *C. diphtheriae *S601 plasmid pNG2 and located downstream of the *tet*(W) gene, whereas a 47-bp stretch of DNA with identity to the 5' end of IS*26 *is present upstream of the *tetLP *gene.

To elucidate the capability of the *tet*(W) gene product to mediate resistance to tetracyclines, including minocycline, the *tet*(W) gene region was amplified by PCR and the resulting DNA fragment was cloned in *E. coli *DH5αMCR into the shuttle vector pEC-K18*mob*2. The recombinant plasmid, designated pKM22, was subsequently transferred into the antibiotic-susceptible host strain *C. glutamicum *ATCC 13032, resulting in *C. glutamicum *KM22. The role of the cloned *tet*(W) gene in tetracycline resistance was examined in *C. glutamicum *KM22 by measuring the MICs of tetracycline and oxytetracycline (first generation tetracyclines), doxycycline and minocycline (second generation tetracyclines), and the atypical tetracycline analog anhydrotetracycline. Additional antimicrobial susceptibility assays served as controls and were performed with *C. resistens *DSM 45100 and *C. glutamicum *ATCC 13032 carrying the empty cloning vector pEC-K18*mob*2. These assays revealed that *C. glutamicum *KM22 gained a remarkable resistance to first and second generation tetracyclines in comparison with the control strain *C. glutamicum *ATCC 13032, displaying MICs from 8 μg ml^-1 ^to 32 μg ml^-1 ^(Table 3). Slightly higher MICs were measured in *C. resistens *DSM 45100 (Table 3). On the other hand, the tested corynebacterial strains revealed the same MIC in the assay with anhydrotetracycline, indicating that the ribosomal protection protein Tet(W) may not confer resistance to this atypical tetracycline analog. This result of the antimicrobial susceptibility assay is obvious as the primary target of anhydrotetracycline is not the bacterial ribosome and the process of translation. The antimicrobial activity of anhydrotetracycline is exerted instead by disrupting bacterial membranes [111,112]. In conclusion, the *tet*(W) gene of pJA144188 is a very likely candidate to confer minocycline resistance in *C. resistens *DSM 45100 and might be responsible for the failure of minocycline therapy in patients with *C. resistens *bacteremia.

**Table 3:** Minimum inhibitory concentrations [μg ml^-1^] of tetracyclines against *C.resistens *and *C. glutamicum*

Strain	Tetracycline	Oxytetracycline	Doxycycline	Minocycline	Anhydrotetracycline
CRES DSM 45100	64	32	16	16	8
CGLU ATCC 13032^a^	0.5	1	1	0.5	8
CGLU KM22	32	32	8	16	8

^a ^*C. glutamicum *ATCC 13032 carrying the empty cloning vector pEC-K18*mob*2

To assess the effect of a subinhibitory concentration of tetracycline (2 μg ml^-1^) on the transcription of *tet*(W) in *C. resistens *DSM 45100, the transcript levels of the *tet*(W) mRNA were determined in induced and non-induced cultures by real-time reverse transcription (RT)-PCR. For this purpose, total RNA samples were purified from *C. resistens *DSM 45100 cultures exposed to 2 μg ml^-1 ^tetracycline for 24 h (induced condition) and control cultures grown in the absence of tetracycline (non-induced condition). Indeed, the transcript level of *tet*(W) was 52-fold higher in the *C. resistens *DSM 45100 culture that has been exposed to tetracycline for 24 h, when compared to the control culture. This data indicated that the expression of the *tet*(W) gene on pJA144188 is regulated at the level of transcription and inducible by tetracycline in *C. resistens *DSM 45100. The respective molecular mechanism is currently unknown, and the role of the putative leader peptide gene in this process, if any, remains to be elucidated. The ribosomal protection gene *tet*(M) from *Staphylococcus aureus *MRSA101 is also inducible by tetracycline at the level of transcription [113]. Expression studies revealed a greatly increased amount of *tet*(M)-specific mRNA when the *S. aureus *cells were first treated with a subinhibitory amount of tetracycline. The *tet*(M) gene was previously also observed by Southern techniques in *C. striatum *strains from clinical specimens [114].

## Conclusions

**Figure 6:**
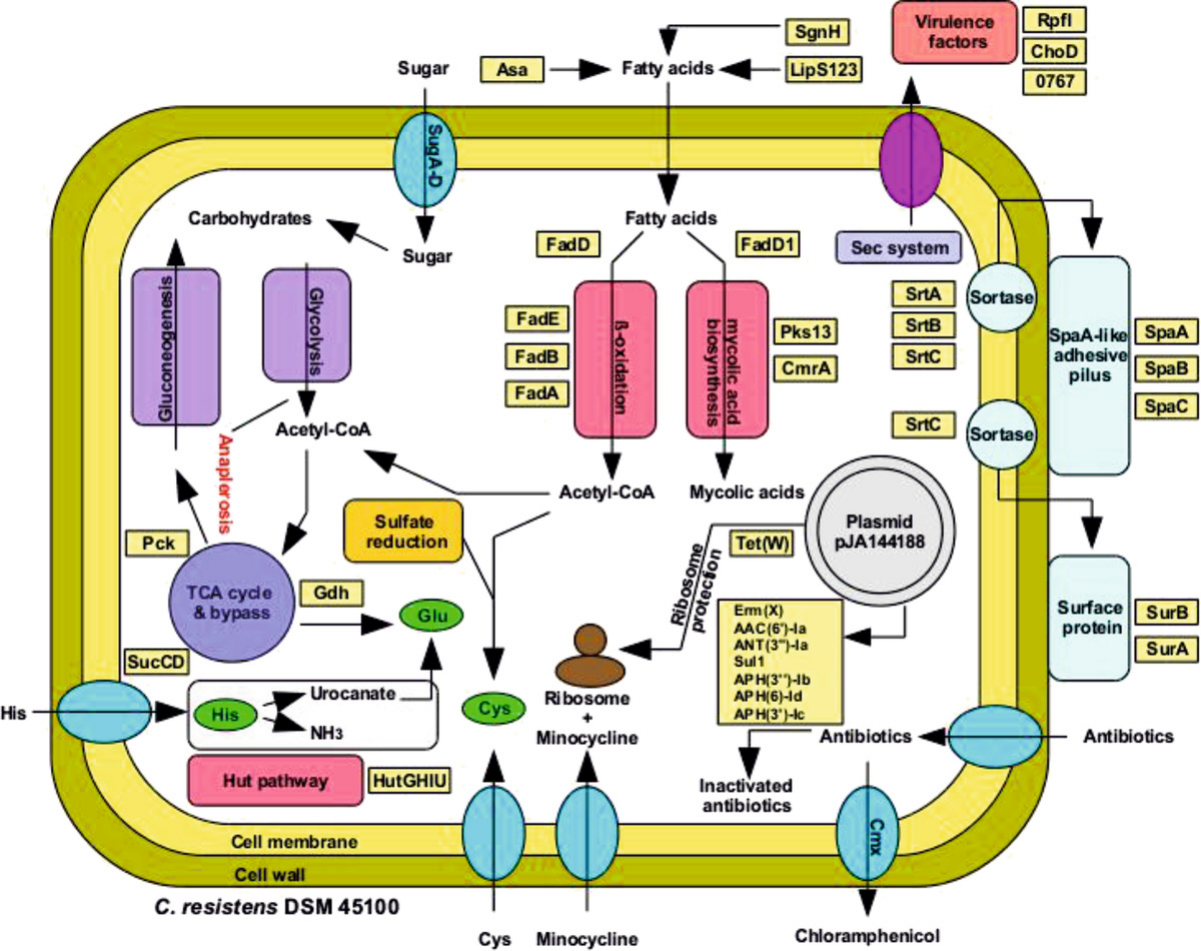
**Overview of prominent metabolic and medically relevant features of *C. resistens *DSM 45100 deduced from the complete genome sequence**. Metabolic features associated with carbohydrate metabolism, histidine utilization, sulfate reduction, fatty acid metabolism, mycolic acid biosynthesis, and pilus formation are shown. Relevant proteins assigned to these processes are labeled by yellow boxes; relevant transport systems are shown as blue circles. The predicted virulence factors are probably secreted by the machinery of the general secretory pathway (Sec system). The role of the predicted virulence factors in ensuring the availability of exogenous fatty acids for growth of *C. resistens *is remarkable. The presence of pJA144188 in *C. resistens *DSM 45100 is indicated and the encoded antibiotic resistance proteins are listed. The role of the Tet(W) protein in ribosomal protection of *C. resistens *DSM 45100 is highlighted, as it is most likely responsible for the clinically relevant resistance to minocycline.

In this study, we describe the complete genome sequence and annotation of the multi-drug resistant clinical isolate *C. resistens *DSM 45100. The sequence analysis revealed comprehensive insights into the metabolic features, virulence functions, and mechanisms for antibiotic resistance of this human pathogen. The integration of these data provides for the first time a detailed view on the deduced lifestyle of *C. resistens *(Figure 6). The strict lipophilic lifestyle of this species is obviously caused by the absence of genes for fatty acid synthesis, sugar uptake, and anaplerotic functions. Therefore, gene loss is the dominant evolutionary mechanism in shaping the metabolic features of *C. resistens*, which are most probably related to the natural habitat. *C. resistens *might colonize of the inguinal or perineal regions of the human body, as these sites of the skin provide elevated amounts of fatty acid substrates for growth by natural secretions that contribute to the formation of hydrolipid films. Moreover, the utilization of L-histidine as a nitrogen or carbon source by enzymes encoded by the *hut *genes suggests that *C. resistens *lives in close proximity to the human genital tract, since the presence of the *hut *pathway is predominantly associated with corynebacteria causing urogenital tract infections. The strict lipophilic lifestyle of *C. resistens *is also linked with enzymatic functions of several predicted virulence factors, which probably ensure the availability of external fatty acids for growth by causing damage to membranes of host cells. Accordingly, the predicted repertoire of candidate virulence factors might explain the low pathogenic potential of *C. resistens*.

The extensive multi-drug resistance of *C. resistens *DSM 45100 is apparently caused by distinct features of chromosomal genes and the presence of plasmid pJA144188. The sequence annotation of pJA144188 provided detailed insights into the gene composition and the modular genetic organization of this plasmid, thereby revealing that horizontal gene transfer represents a key factor in the development of multi-drug resistance in *C. resistens*. A similar set of antibiotic resistance genes is present in other multi-drug resistant corynebacteria, such as *C. striatum, C. urealyticum*, and *C. jeikeium*. This collection includes the *erm*(X) and *cmx *genes, as well as *aphA1-IAB *and the *strA*-*strB *tandem gene pair [24,25,115]. The *tet*(W) gene of pJA144188 encodes a ribosomal protection protein that confers resistance to first and second generation tetracyclines, including the clinically relevant tetracycline derivative minocycline. The presence of the *tet*(W) gene on pJA144188 has tremendous impact on the treatment of human infections associated with *C. resistens*, as the cross-resistance profile of the Tet(W) protein can contribute to the failure of minocycline therapies in (immunocompromised) patients.

## Methods

### Bacterial strains and growth conditions

*C. resistens *DSM 45100 (GTC 2026, CCUG 50093) was obtained as a lyophilized culture from DSMZ (Braunschweig, Germany) and routinely grown on solid BYT medium at 37°C [116]. This clinical isolate was originally recovered from a positive blood culture taken from a patient with acute myelocytic leukemia and initially named SICGH 158 [14]. *E. coli *DH5αMCR was used for standard cloning procedures and cultured on Luria-Bertani medium at 37°C [117]. The wild-type strain *C. glutamicum *ATCC 13032 (American Type Culture Collection, Manassas, VA) was routinely grown at 30°C in CGXII minimal medium containing 30 μg l^-1 ^protocatechuic acid and 420 μg l^-1 ^thiamine [118]. Kanamycin was used for the selection of plasmids in *E. coli *(50 μg ml^-1^) and *C. glutamicum *(25 μg ml^-1^). The growth of shake-flask cultures was monitored by measuring the optical density at 600 nm with an Eppendorf BioPhotometer.

### Genome sequencing of *C. resistens *DSM 45100

Genomic DNA of *C. resistens *DSM 45100 was purified by an alkaline lysis procedure [119] from 20-ml aliquots of an overnight culture grown in liquid BYT medium supplemented with 1.25% (w/v) glycine. The original lysis protocol was modified as follows: (i) The *C. resistens *cells were incubated in a 30 mg ml^-1 ^lysozyme solution at 37°C for 1 h. (ii) The harvested cells were lysed in 0.7 ml 10% (w/v) SDS solution at 37°C for 15 min. A total of 5 μg of purified genomic DNA from *C. resistens *DSM 45100 was used for constructing a single-stranded template DNA library. The preparation and sequencing of the DNA library were performed according to standard protocols from Roche Applied Science. The Genome Sequencer FLX System and Titanium chemistry (Roche Applied Science) were applied for sequencing of the genomic DNA. The sequence reads were assembled with the GS Assembler Software (version 2.3).

The remaining gaps in the genome sequence of *C. resistens *DSM 45100 were closed by PCR with Phusion hot start high-fidelity DNA polymerase (Finnzymes) and genomic template DNA. All primers used in this study were synthesized by Metabion. The PCR assays were carried out with a TProfessional PCR thermocylcer (Biometra) according to standard protocols (Finnzymes). The amplified DNA fragments linking the individual contigs were sequenced by IIT Biotech. Chromosomal DNA sequences and plasmid sequences were uploaded separately into the Consed program [16] to generate the complete genome sequence of *C. resistens *DSM 45100.

### Annotation and bioinformatic analysis of the genome sequence

The assembled sequences of *C. resistens *DSM 45100 were uploaded into the bacterial genome annotation system GenDB [17]. The automatic annotation of the complete genome sequence was performed as described previously [25], followed by manual curation of the data. The genome plot of *C. resistens *DSM 45100 was generated with the web tool DNAPlotter [120]. The origin of chromosomal replication of *C. resistens *was predicted with the web version of the Ori-Finder tool [20]. Clustered regularly interspaced short palindromic repeats (CRISPRs) were detected with the CRISPRFinder tool [30]. Analyses of the predicted gene content and the metabolic properties of *C. resistens *were accomplished by the software tools EDGAR [22] and CARMEN [121], using their default parameters. The synteny between the chromosome of *C. resistens *DSM 45100 and that of *C. jeikeium *K411 was calculated by the EDGAR software [22].

The annotated sequence of the *C. resistens *DSM 45100 chromosome has been deposited in the GenBank database with accession number CP002857 and is available from the RefSeq database with accession number NC_015673. The sequence of plasmid pJA144188 is available from GenBank with accession number FN825254 and from RefSeq with accession number NC_014167.

### Antimicrobial susceptibility assays with tetracyclines and fluoroquinolones

The antimicrobial susceptibilities of *C. resistens *DSM 45100, *C. glutamicum *ATCC 13032 and *C. glutamicum *KM22 were determined *in vitro *by a macrobroth dilution method according to the guidelines of the Clinical and Laboratory Standards Institute [122]. The antibiotics tetracycline, oxytetracycline, doxycycline, minocycline, and anhydrotetracycline as well as the fluoroquinolones ciprofloxacin, danofloxacin, levofloxacin, norfloxacin, and sparfloxacin were purchased from Sigma-Aldrich. All antibiotics were tested *in vitro *in the range of 0.1 to 256 μg ml^-1^. The corynebacterial cells were grown in Mueller-Hinton broth (Merck) supplemented with 1% (v/v) Tween 80. The minimum inhibitory concentration (MIC) was taken as the lowest concentration of the antibiotic to completely inhibit the visible growth of the bacteria after incubation for 24 h at 37°C [122].

### Cloning of the *tet*(W) gene from *C. resistens *DSM 45100

The preparation of plasmid DNA from *E. coli *DHαMCR cells was performed by an alkaline lysis technique using the QIAprep Spin Miniprep Kit (Qiagen). The protocol was modified for *C. glutamicum *cells by using 20 mg ml^-1 ^lysozyme in resuspension buffer P1 and by incubating the assay at 37°C for 3 h. DNA restriction, DNA analysis by agarose gel electrophoresis, and DNA ligation were performed according to standard procedures [117]. The transformation of plasmid DNA was carried out by electroporation using electrocompetent *E. coli *and *C. glutamicum *cells [123,124]. The *tet*(W) gene was amplified by PCR with the primer pair tet(W)fwd (GATCTAG-GATCCGTGCGGGGAAGAAAAAT) and tet(W)rev (GATCTATCTAGACGCAATAGCCAG-CAATGA). The amplified *tet*(W) gene was cloned in *E. coli *DHαMCR into the shuttle vector pEC-K18*mob*2 [124], resulting in plasmid pKM22. DNA of pKM22 was isolated from *E. coli *and subsequently transferred into *C. glutamicum *ATCC 13032, leading to strain *C. glutamicum *KM22.

### RNA techniques and measurement of *tet*(W) transcript levels

The isolation and purification of total RNA from *C. resistens *DSM 45100 cultures was carried out as described previously [125]. The strain was grown in BYT medium without tetracycline (non-induced) and in BYT medium supplemented with 2 μg ml^-1 ^tetracycline (induced). The transcript levels of the *tet*(W) gene were measured by real-time reverse transcriptase PCR (RT-PCR) with the LightCycler instrument (Roche Applied Science), using the SensiMix One-Step Kit (Quantace) and the primer pair tet(W)LC1 (TTCGATGGTGGCACAGTA) and tet(W)LC2 (TTGTTCGGCTGGAACGTA). The differences in *tet*(W) transcript levels between induced and non-induced cultures of *C. resistens *DSM 45100 were determined by comparing the crossing points of two biological samples, each measured with two technical replicates. Crossing points were calculated by the LightCycler software (Roche Applied Science). The measured crossing point (CP) is the cycle at which PCR amplification begins its exponential phase and is considered the point that is most reliably proportional to the initial RNA concentration. The relative change in *tet*(W) transcript levels was determined as 2^-ΔCP^, with ΔCP being equal to the difference of the measured crossing points for the test (induced) and the control (non-induced) condition. The quality of the measurements was ensured by melting curve analysis with the LightCycler software (Roche Applied Science).

## Authors' contributions

JAS sequenced and annotated the genome of *C. resistens *and prepared the manuscript. IM supported the gap closure process and the annotation of the chromosome. KM participated in annotating plasmid pJA144188 and carried out the functional analysis of *tet*(W). SW contributed to the manual annotation of the genome sequence and the quinolone-resistance-determining region. JB implemented the EDGAR project for cluster 3 corynebacteria. SJ provided support for the GenDB project. JES prepared the GenBank file and provided bioinformatic support. ET and AT participated in data evaluation. All authors read and approved the final version of the manuscript.

## Supplementary Material

Additional file 1 **Annotation of pathways involved in central carbohydrate metabolism of *C. resistens *DSM 45100**. The PDF contains a metabolic reconstruction based on manually curated pathway maps related to the central carbohydrate metabolism. Click here for file

Additional file 2 **Annotation of pathways involved in amino acid metabolism of *C. resistens *DSM 45100**. The PDF contains a metabolic reconstruction based on manually curated pathway maps related to the uptake and metabolism of amino acids. Click here for file
