# Supplementary material for: Complete genome sequence, lifestyle, and multi-drug resistance of the human pathogen Corynebacterium resistens DSM 45100 isolated from blood samples of a leukemia patient
Source: BMC Genomics. 2012 Apr 23;13:141. doi: 10.1186/1471-2164-13-141 (PMC3350403; doi:10.1186/1471-2164-13-141)
Supplement: Additional file 1 — Annotation of pathways involved in central carbohydrate metabolism of C. resistens DSM 45100. The PDF contains a metabolic reconstruction based on manually curated pathway maps related to the central carbohydrate metabolism. [file 1471-2164-13-141-S1.PDF]

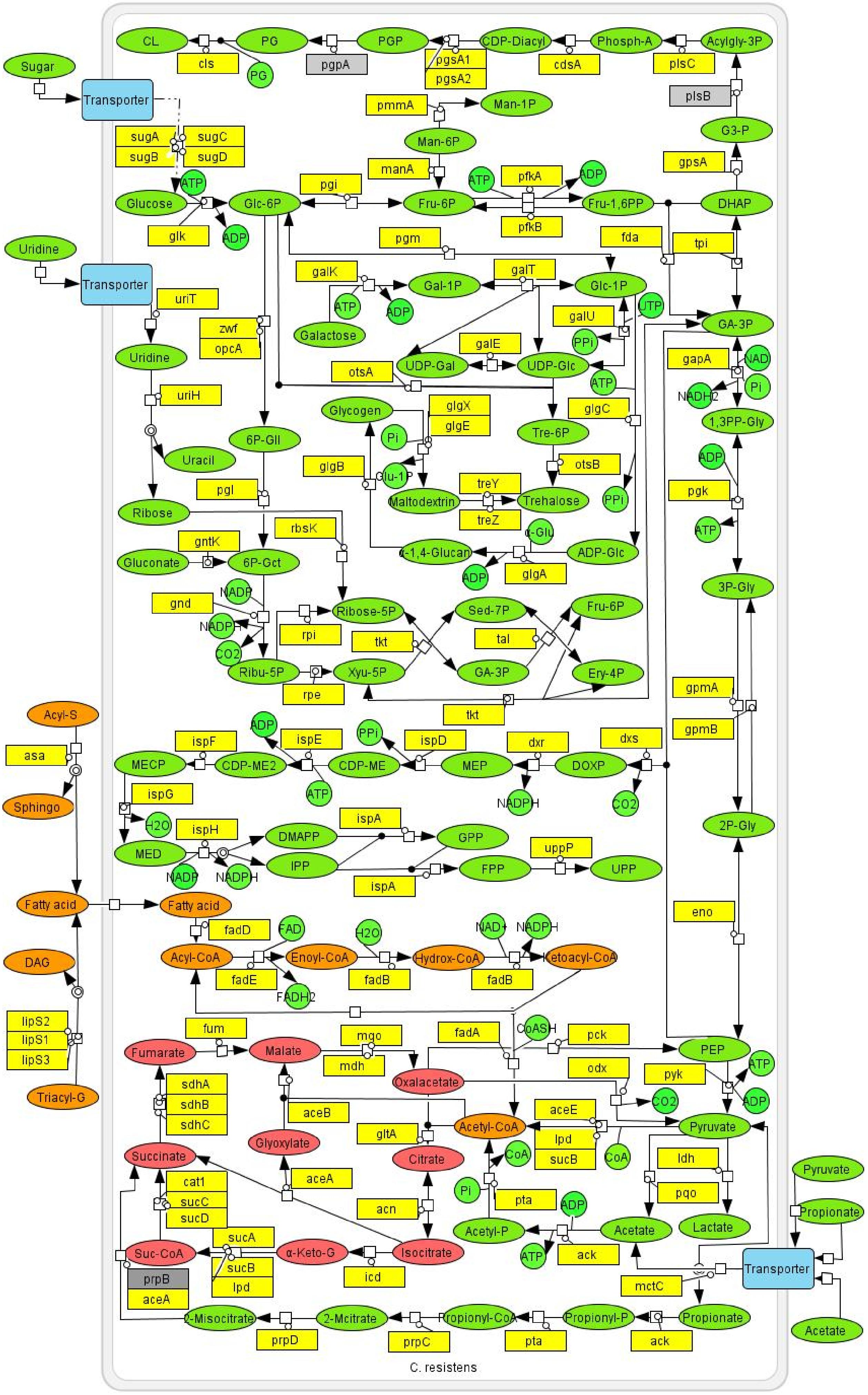
CL
PG
PGP
CDP-Diacyl
Phosph-A
Acylgly-3P
cls
PG
pgpA
pgsA1
pgsA2
cdsA
plsC
Sugar
Transporter
plsB
pmmA
Man-1P
G3-P
sugA
sugC
sugB
sugD
Man-6P
gpsA
manA
ATP
pfkA
ADP
pgi
ATP
Glucose
Glc-6P
Fru-6P
Fru-1,6PP
DHAP
glk
ADP
pfkB
Uridine
pgm
fda
tpi
Transporter
galK
galT
uriT
Gal-1P
Glc-1P
ATP
ADP
UTP
zwf
opcA
Galactose
galU
GA-3P
galE
PPi
Uridine
otsA
UDP-Gal
UDP-Glc
gapA
NAD
uriH
ATP
Pi
NADH2
Glycogen
glgX
glgC
1,3PP-Gly
glgE
Tre-6P
Pi
Uracil
6P-Gll
ADP
glgB
otsB
Glu-1P
pgl
treY
pgk
Maltodextrin
Trehalose
PPi
Ribose
treZ
ATP
rbsK
α-Glu
gntK
α-1,4-Glucan
ADP-Glc
Gluconate
6P-Gct
ADP
glgA
3P-Gly
NADP
gnd
Sed-7P
Fru-6P
Ribose-5P
NADPH
rpi
tkt
tal
CO2
Ribu-5P
Xyu-5P
GA-3P
Ery-4P
rpe
gpmA
Acyl-S
tkt
gpmB
ADP
ispF
ispE
PPi
ispD
dxr
dxs
asa
MECP
CDP-ME2
CDP-ME
MEP
DOXP
ATP
Sphingo
ispG
NADPH
CO2
H2O
ispA
ispH
DMAPP
GPP
2P-Gly
uppP
MED
IPP
FPP
UPP
NADP
NADPH
ispA
Fatty acid
Fatty acid
eno
fadD
FAD
H2O
NAD+
NADPH
DAG
Acyl-CoA
Enoyl-CoA
Hydrox-CoA
Ketoacyl-CoA
fadE
fadB
fadB
FADH2
lipS2
lipS1
lipS3
fum
mqo
fadA
CoASH
pck
Fumarate
Malate
PEP
mdh
odx
ATP
Triacyl-G
Oxalacetate
pyk
sdhA
sdhB
CO2
ADP
sdhC
aceB
aceE
gltA
Acetyl-CoA
Pyruvate
lpd
Glyoxylate
Succinate
CoA
sucB
CoA
Citrate
ldh
cat1
Pyruvate
aceA
Pi
pqo
sucC
pta
sucD
acn
ADP
Propionate
Acetyl-P
Acetate
Lactate
sucA
Suc-CoA
α-Keto-G
Isocitrate
ATP
ack
Transporter
sucB
prpB
icd
lpd
mctC
aceA
2-Misocitrate
2-Mcitrate
Propionyl-CoA
Propionyl-P
Propionate
Acetate
prpD
prpC
pta
ack
C. resistens

Additional file 1 Reconstruction of pathways involved in the central metabolism of *C. resistens* DSM 45100. The metabolic reconstruction was performed with manually curated pathway maps in conjunction with the bioinformatic tool CARMEN and the CellDesigner software. Metabolites are represented by ovals, whereas orange ones are involved in fatty acid metabolism and red ones are involved in the TCA cycle. Corresponding genes are displayed in yellow boxes, grey genes are lacking in the *C. resistens* genome sequence. Abbreviations for metabolites are as follows: Acetyl-P, acetyl phosphate; Acyl-CoA, acyl-Coenzyme A; Acylgly-3P, 1-acyl-glycerol-3-phosphate; Acyl-S, acyl-sphingosine; ADP, adenosine diphosphate; ATP, adenosine triphosphate; ADP-Glc, ADP-glucose; CDP-Diacyl, CDP-diacylglycerol; CDP-ME, 4-cytidyl diphospho-2*C*-methyl-D-erythritol; CDP-ME2, 4-cytidyl diphospho-2*C*-methyl-D-erythritol 2-phosphate; CL, cardiolipin; CoA/CoASH, Coenzyme A; DAG, diacylglycerol; DHAP, dihydroxyacetone phosphate; DMAPP, dimethylallyl diphosphate; DOXP, 1-deoxy-D-xylulose-5-phosphate; Enoyl-CoA, enoyl-Coenzyme A; Ery-4P, erythrose-4-phosphate; FAD, flavin-adenine-dinucleotide; FPP, farnesyl pyrophosphate; Fru-1,6PP, fructose-1,6-bisphosphate; Fru-6P, fructose-6-phosphate; Gal-1P, galactose-1-phosphate; GA-3P, glyceraldehyde-3-phosphate; Glc-1P, glucose-1-phosphate; Glc-6P, glucose-6-phosphate; GPP, geranyl pyrophosphate; G3-P, glycerol-3-phosphate; Hydrox-CoA, hydroxyacyl-Coenzyme A; Hydrox-CoA, hydroxyacyl-Coenzyme A; IPP, isopentenyl pyrophosphate; Ketoacyl-CoA, ketoacyl-Coenzyme A; Man-1P, mannose-1-phosphate; Man-6P, mannose-6-phosphate; MECP, methyl-erythritol-2,4-cycloPP; MED, hydroxyl-2-methyl-2-butenyl-4-PP; MEP, 2-*C*-methyl-D-erythritol 4-phosphate; NAD/NADH2, nicotinamide adenine dinucleotide; NADP/NADPH, nicotinamide adenine dinucleotide phosphate; PEP, phosphoenolpyruvate; PG, phosphoglycolate; PGP, 2-phosphoglycolate; Phosph-A, phosphatidate; Pi, phosphate; PPi, pyrophosphate; Propionyl-CoA, propionyl-Coenzyme A; Propionyl-P, propionyl phosphate; Ribose-5P, ribose-5-phosphate; Ribu-5P, ribulose-5-phosphate; Sed-7P, sedoheptulose-7-phosphate; Sphingo, sphingosine; Suc-CoA, succinyl-Coenzyme A; Tre-6-P, trehalose-6-phosphate; Triacyl-G, triacylglycerol; UDP-Gal, UDP-galactose; UDP-Glc, UDP-glucose; UPP, undecaprenyl pyrophosphate; Xyu-5P, xylulose-5-phosphate; 1,3PP-Gly, 1,3-bisphosphoglycerate; 2-Mcitrate, 2-methylcitrate; 2-Misocitrate, 2-methylisocitrate; 2P-Gly, 2-phosphoglycerate; 3P-Gly, 3-phosphoglycerate; 6P-Gct, 6-phosphogluconate; 6P-Gll, 6-phosphogluconolactone; α-keto-G; α-ketoglutarate; α-1,4-Glucan, α(1-4)glucan.
