# Supplementary material for: Complete genome sequence, lifestyle, and multi-drug resistance of the human pathogen Corynebacterium resistens DSM 45100 isolated from blood samples of a leukemia patient
Source: BMC Genomics. 2012 Apr 23;13:141. doi: 10.1186/1471-2164-13-141 (PMC3350403; doi:10.1186/1471-2164-13-141)
Supplement: Additional file 2 — Annotation of pathways involved in amino acid metabolism of C. resistens DSM 45100. The PDF contains a metabolic reconstruction based on manually curated pathway maps related to the uptake and metabolism of amino acids. [file 1471-2164-13-141-S2.PDF]

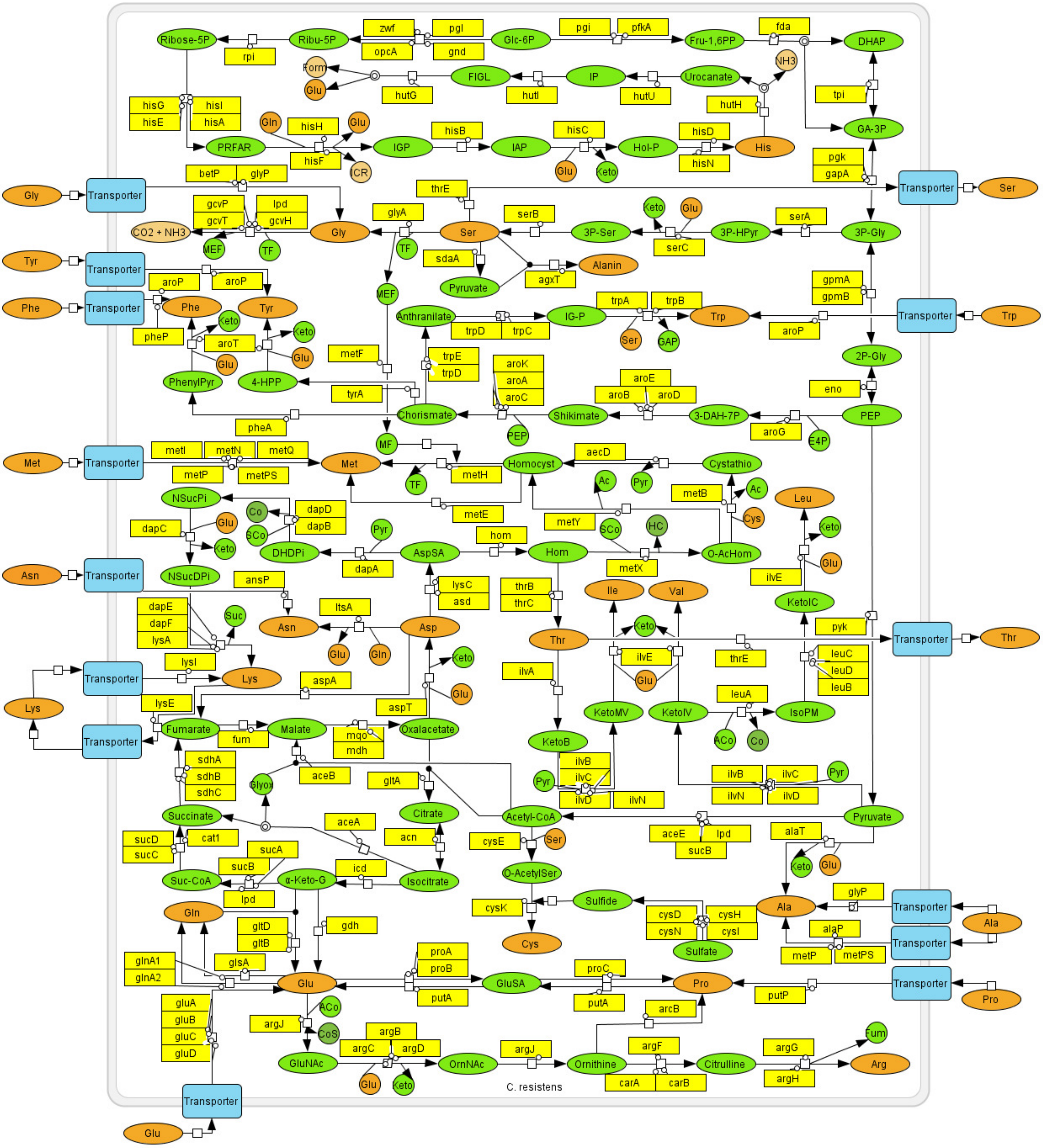

Ribose-5P
rpi
Ribu-5P
zwf
opcA
pgl
gnd
Glc-6P
pgi
pfkA
Fru-1,6PP
fda
DHAP
Form
Glu
hutG
FIGL
hutI
IP
hutU
Urocanate
NH3
hutH
tpi
GA-3P
hisG
hisE
hisI
hisA
Gln
hisH
hisF
Glu
ICR
PRFAR
IGP
hisB
IAP
hisC
Glu
Keto
Hol-P
hisD
hisN
His
pgk
gapA
Gly
Transporter
betP
glyP
thrE
Transporter
Ser
gcvP
gcvT
lpd
gcvH
CO2 + NH3
MEF
TF
Gly
glyA
TF
Ser
serB
3P-Ser
Keto
Glu
serC
3P-HPyr
serA
3P-Gly
Tyr
Transporter
aroP
aroP
sdaA
Pyruvate
agxT
Alanin
MEF
Phe
Transporter
Phe
Tyr
Keto
Keto
pheP
aroT
Glu
Glu
PhenylPyr
4-HPP
Anthranilate
trpD
trpC
IG-P
trpA
trpB
Ser
GAP
Trp
aroP
gpmA
gpmB
Transporter
Trp
metF
trpE
trpD
tyrA
aroK
aroA
aroC
aroE
aroB
aroD
2P-Gly
eno
Chorismate
Shikimate
3-DAH-7P
PEP
pheA
PEP
aroG
E4P
Met
Transporter
metI
metN
metQ
metP
metPS
Met
MF
TF
metH
Homocyst
aecD
Cystathio
Ac
Pyr
metB
Ac
Cys
Leu
NSucPi
Co
dapD
dapB
dapC
Glu
SCo
Keto
Pyr
DHDPi
dapA
AspSA
hom
Hom
metY
SCo
HC
metX
O-AcHom
Keto
Glu
ilvE
Asn
Transporter
NSucDPi
ansP
lysC
asd
thrB
thrC
Ile
Val
KetoIC
dapE
dapF
lysA
Suc
ltsA
Asn
Asp
Keto
pyk
Transporter
Thr
Glu
Gln
Thr
ilvE
Glu
thrE
leuC
leuD
leuB
lysI
Lys
Transporter
Lys
aspA
Keto
Glu
ilvA
lysE
aspT
leuA
Transporter
Fumarate
fum
Malate
mqo
mdh
Oxalacetate
KetoMV
KetoIV
IsoPM
ACo
Co
KetoB
sdhA
sdhB
sdhC
Glyox
aceB
gltA
ilvB
ilvC
ilvD
Pyr
ilvN
ilvB
ilvN
ilvC
ilvD
Pyr
Succinate
aceA
Citrate
acn
Acetyl-CoA
Pyruvate
cysE
Ser
aceE
lpd
sucB
alaT
sucD
sucC
cat1
sucA
sucB
icd
Keto
Glu
Suc-CoA
α-Keto-G
Isocitrate
O-AcetylSer
lpd
Gln
gltD
gltB
gdh
cysK
Sulfide
cysD
cysN
cysH
cysI
Ala
glyP
Transporter
Ala
Cys
Sulfate
alaP
Transporter
metP
metPS
glnA1
glnA2
glsA
proA
proB
Glu
GluSA
proC
Pro
putA
putA
putP
Transporter
Pro
gluA
gluB
gluC
gluD
argJ
ACo
CoS
arcB
GluNAc
argB
argC
argD
OrnNAc
argJ
Ornithine
argF
Citrulline
argG
Fum
Arg
Glu
Keto
carA
carB
argH
C. resistens
Transporter
Glu

Additional file 2 Reconstruction of pathways involved in amino acid metabolism of *C. resistens* DSM 45100. The metabolic reconstruction was performed with manually curated pathway maps in conjunction with the bioinformatic tool CARMEN and the CellDesigner software. Metabolites are represented in ovals, mature amino acids are colored in orange. Corresponding genes are displayed in yellow boxes. Abbreviations for metabolites are as follows: Ac, acetate; ACo, acetyl-Coenzyme A; Ala, alanine; Arg, arginine; Asn, asparagine; Asp, aspartic acid; AspSA, aspartate semialdehyde; CoA/CoASH, Coenzyme A; Cys, cysteine; Cystathio, L,L-cystathionine; DHAP, dihydroxyacetone phosphate; DHDPi, dihydrodipicolinate; FIGL, formimino-glutamate; Fru-1,6PP, fructose-1,6-bisphosphate; Fum, fumarate; GA-3P/GAP, glyceraldehyde-3-phosphate; Glc-6P, glucose-6-phosphate; Gln, glutamine; Glu, glutamic acid; GluNAc, *N*-acetylglutamate; GluSA, γ-glutamic semialdehyde; Gly, glycin; Glyox, glyoxalate; His, histidine; Hol-P, L-histidinol phosphate; Hom, homoserine; Homocyst, homocysteine; IAP, imidazole acetol phosphate; IGP, imidazole glycerol phosphate; IG-P, indole glycerol phosphate; Ile, isoleucine; IP, imidazolone propionate; IsoPM, α-isopropylmalate; KetoB, α-ketobutyrate; KetoIC, α-ketoisocaproate; KetoIV, α-ketoisovalerate; KetoMV, α-keto-β-methylvalerate; Leu, leucine; Lys, lysine; Met, methionine; NSucDPi, *N*-succinyldiaminopimelate; NSucPi, *N*-succinyl-α-amino-ε-keto-pimelate; O-AcetylSer, *O*-acetylserine; O-AcHom, *O*-succinylhomoserine; OrnNAc, *N*-acetylornithine; PEP, phosphoenolpyruvate; Phe, phenylalanine; Phenylpyr, phenylpyruvate; PRFAR, phosphoribosyl formimino-5-aminoimidazole-4-carboxamide ribonucleotide; Pro, proline; Pyr, pyruvate; Ribose-5P, ribose-5-phosphate; Ribu-5P, ribulose-5-phosphate; Ser, serine; Suc-CoA/SCo, succinyl-Coenzyme A; Thr, threonine; Trp, tryptophan; Tyr, tyrosine; Val, valine; 2P-Gly, 2-phosphoglycerate; 3-DAH-7P, 3-deoxy-D-arabino-heptulosonate-7-phosphate; 3P-HPyr, 3-phosphohydroxypyruvate; 3P-Gly, 3-phosphoglycerate; 3P-Ser, 3-phosphoserine; 4-HPP, 4-hydroxyphenylpyruvate; α-keto-G/Keto; α-ketoglutarate.
